# How media presence triggers participation in citizen science—The case of the mosquito monitoring project ‘Mückenatlas‘

**DOI:** 10.1371/journal.pone.0262850

**Published:** 2022-02-17

**Authors:** Nadja Pernat, Jana Zscheischler, Helge Kampen, Emu-Felicitas Ostermann-Miyashita, Jonathan M. Jeschke, Doreen Werner

**Affiliations:** 1 Leibniz Centre for Agricultural Landscape Research (ZALF), Müncheberg, Germany; 2 Department of Biology, Chemistry, Pharmacy, Institute of Biology, Freie Universität, Berlin, Germany; 3 Berlin-Brandenburg Institute of Advanced Biodiversity Research (BBIB), Berlin, Germany; 4 Friedrich-Loeffler-Institut, Federal Research Institute for Animal Health, Greifswald–Insel Riems, Germany; 5 Thaer-Institute of Agricultural and Horticultural Sciences, Humboldt-Universität zu, Berlin, Germany; 6 Leibniz Institute of Freshwater Ecology and Inland Fisheries (IGB), Berlin, Germany; Instituto Federal de Educacao Ciencia e Tecnologia Goiano - Campus Urutai, BRAZIL

## Abstract

Since 2012, the citizen science project ‘Mückenatlas’ has been supplementing the German mosquito monitoring programme with over 28,000 submissions of physical insect samples. As the factors triggering people to catch mosquitoes for science are still unknown, we analysed the influence of mass media reports on mosquito submission numbers. Based on a theoretical framework of how mass media affect citizen responsiveness, we identified five possible influencing factors related to citizen science: (i) project awareness and knowledge, (ii) attention (economy), (iii) individual characteristics of citizen scientists and targeted communication, (iv) spatial differences and varying affectedness, and (v) media landscape. Hypotheses based on these influencing factors were quantitatively and qualitatively tested with two datasets: clipping data of mass media reports (online, television, radio and print) referring to or focussing on the ‘Mückenatlas’, and corresponding data of ‘Mückenatlas’ submissions between 2014 and 2017. In general, the number of media reports positively affected the number of mosquito submissions on a temporal and spatial scale, i.e. many media reports provoke many mosquito submissions. We found that an already heightened public and media awareness of mosquito-relevant topics combined with a direct call-to-action in a media report title led to a maximum participation. Differences on federal state level, however, suggest that factors additional to quantitative media coverage trigger participation in the ‘Mückenatlas’, in particular the mosquito affectedness of the resident population. Lastly, media types appear to differ in their effects on the number of submissions. Our results show under which circumstances the media presence of the ’Mückenatlas’ is most effective in activating people to submit mosquito samples, and thus provide advice for designing communication strategies for citizen science projects.

## 1. Introduction

With continuing outbreaks of mosquito-borne diseases in Mediterranean countries [1] as well as recent cases of West Nile fever as far north as Germany [2, 3], management of vector-competent mosquitoes has become an important political and scientific issue throughout Europe. In 2011, mosquito research returned to the scientific agenda in Germany in the form of a nationwide monitoring programme, aiming at gaining knowledge about the occurrence and distribution of native and non-native mosquito species [4]. Implemented in this programme is the citizen science project ‘Mückenatlas’ (German for ‘mosquito atlas’), one of the longest running and most successful citizen science projects in Germany [5].

The ‘Mückenatlas’ is hosted at two institutions, the Leibniz Centre for Agricultural Landscape Research (ZALF) and the Friedrich-Loeffler-Institut (FLI), with up to four people working on the sample identification, reference collection, participant support and database maintenance (S1 Fig in S2 File). Within the project, citizens are asked to catch mosquitoes (wherever and whenever they want to), to kill them by freezing, to fill out a form downloadable from the project’s website (www.mueckenatlas.com) or available at the project’s administrative office (S1 File), and submit both the mosquito(es) and the form to the involved research institutions. There, the mosquito sample is determined to species level and the achieved information entered into the German mosquito database, CULBASE.

The participants receive an individual letter or email with feedback about the catch and, optionally, a marker with their name or a pseudonym on the collectors’ map of the website. In return, the website is regularly updated with research results achieved on the basis of the submissions. After eight years of operation, the ‘Mückenatlas’ has received more than 28,000 submissions, accumulating to close to 154,000 submitted specimens by June 2021.

The ‘Mückenatlas’ was launched in 2012 at a time when citizen science–the involvement of the public in scientific research–globally gained momentum. Although public participation in biodiversity monitoring has a long tradition [6–8], it is barely a decade ago when citizen science has become widespread in the scientific community [9–11] and evolved from a mere method to a research subject itself [12–14]. An important reason for the rise of citizen science are web-enabled sophisticated electronic devices, such as smartphones, which facilitate participation in research projects by collecting or processing data [15]. Since 2010, funding programmes have been launched worldwide, citizen science hubs formed and cooperations across national borders established [16], for example through continental citizen science associations (e.g. the European Citizen Science Association, ECSA) or the founding of the only subject-specific journal to date [17]. At the same time, the scientific discourse about advantages and drawbacks of this emerging scientific discipline is in full swing [18–23].

Due to their familiarity through direct interaction with humans (e.g. biting, buzzing) and their potential health risk through the transmission of pathogens, mosquitoes are highly appropriate subjects for citizen science. Indeed, mosquito-related projects are finished, running or emerging across the globe [24–27]. Yet, in contrast to the ’Mückenatlas’, the majority of projects are limited to invasive mosquitoes [28, 29] and are designed for a short period of time [30, 31]. Some projects make use of a smartphone app, such as the originally Spanish ’Mosquito Alert’ [32], which was launched in several European countries in 2020, or the Italian ’ZanzaMapp’ for measuring mosquito nuisance [33]. Others apply professional traps operated by volunteers [28, 34] or work with physical samples like the ‘Mückenatlas’ [35].

While knowledge about mosquito phenology and distribution in Germany has vastly increased due to the citizens’ involvement [4, 36], the reasons why people participate are still subject to speculation. Only few studies have been conducted on the individuals’ initial motivation to start participating in a citizen science project (but see [37] for online citizen science projects). According to the literature on environmental volunteers, the decision for participation is influenced by the motivation to take part, the personal background that must fit to the project and the basic condition that the project is known to the potential participant [38, 39].

With regard to the latter, social and traditional media play a key role for both initial and sustaining participation in citizen science [40], but only limited studies exist that connect media coverage with participation rates [41–44]. The media particularly trigger initial participation, by drawing attention to the project either through a timely limited campaign in a concerted approach of traditional and social media channels or through continuous reporting through mass media [41, 44, 45]. Consistent and continuous reporting presents the project’s scientists as trustworthy and accessible experts to the public [42, 44], which, in turn, results in further enquiries from the press.

Active contacts to the media have been established since the beginning of the ‘Mückenatlas’, especially with the *Deutsche Presseagentur* (German Press Agency, the largest news agency in Germany, in the following abbreviated as ‘dpa’) as a content provider that triggers print and online media reports. Hecker et al. [46] already speculated that the media is one of four main drivers for effective communication with citizens and a crucial factor for the success of the ‘Mückenatlas’ in terms of attracting attention and spreading news about the project. Despite this potential relevance, the role of the media or the connection between media coverage and the activation of citizens to participate in the project, respectively, has not been studied so far. Knowledge about this interrelation could help future projects to design more targeted media communication to attract potential citizen scientists.

## 2. Studying how participation in citizen science projects relates to media presence—A theoretical framework

Communication via traditional mass media is a frequently used approach to attract participants and draw attention to citizen science projects [43, 44]. However, little is known about the effective design of such communication processes with regard to the activation and successful participation of citizen scientists. Communication theories assume that the effectiveness of a mass communication depends on numerous influencing factors [47]. Thus, there are a number of different, but complementary communication models, each addressing other variables for understanding different forms of communication (for an overview see [48]).

For our analysis, we had to reduce this complexity and focus on the most important variables and their relationships in terms of convincing citizen scientists to participate in our project. Media effects research mainly examines the relationships between input variables (e.g., media information and its characteristics, recipient situation, etc.) and output variables (e.g., attitudes, beliefs, behaviour). In this context, the communication matrix model of persuasion developed by McGuire (1989) [49] has become enormously influential. The matrix model integrates different theoretical approaches of mass media communication with a ladder of different response steps demonstrating that effective communication requires a whole sequence of steps in an effect chain.

Based on this persuasion matrix model and a literature review of research on mass media effects, we identified relevant factors for the responsiveness/resonance to mass communication events in citizen science projects: (i) project awareness and knowledge, (ii) attention (economy), (iii) individual characteristics of citizen scientists and targeted communication, (iv) spatial differences and affectedness and (v) media landscape. ‘Project awareness and knowledge’ and ‘attention (economy)’ represent the first two steps of persuasion in McGuire’s matrix model. They are thus preconditions for the response rate of citizen scientists. While they constitute output variables of the communication process, the other three categories include input variables related to the individual recipients’ situation (‘individual characteristics of citizen scientists’), the message design (‘targeted communication’) and the source of messages (‘media landscape’).

- Project awareness and knowledge: Mass media raise awareness, communicate knowledge and can play a mediating role between citizen engagement and attention to socially relevant issues (e.g. [50]). Thus, public awareness and knowledge about a citizen science project is a precondition to convince citizen scientists to participate in a project [38, 39]. It may thus be assumed that the level of awareness of a project influences the participation/response rate of citizen scientists [44]. Following the persuasion matrix model, the first step in the media effect chain is ‘exposure to a message’. As a consequence, we hypothesize that a higher number of media reports leads to a higher participation/response rate, and in the case of the ‘Mückenatlas’ to a higher number of mosquito submissions on a temporal and spatial scale.
- Attention (economy): The next step of persuasion in McGuire’s matrix model is ‘attention’. The capacities of media recipients to receive and process information are limited [51] and mass media communication is always selective regarding perception, interpretation and understanding. Similarly, transmission times and space for texts in print media are finite. Mass media have a gatekeeping function and shape reaching potential citizen scientists [52]. Thus, communication actions of citizen science projects always compete for attention with other events of public interest. Regional elections or other emerging incidents can positively or negatively affect the attention given to the project which may result in different citizen participation rates [44, 53]. Therefore, we looked for a possible link between increased media attention to mosquito-related topics and participation rates in the ‘Mückenatlas’.
- Individual characteristics of citizen scientists and targeted communication: There is a number of personal characteristics of citizens that affect the motivation to participate [54–56]. While some of these factors cannot be basically influenced, such as general knowledge, cognitive capacities, curiosity or the general attitude towards science, others may be stimulated by a targeted communication including emotional language that addresses citizen’s situations and every-day life. Resonance occurs when a topic has meaning for the recipient [57]. This meaning arises for the recipient when he or she can integrate the information received into his or her world of experience. Thus, we hypothesize that (temporal) differences in response rates to media coverage may not only be explained quantitatively, but partly also by qualitative differences in media coverage referring to language style, framing and contextualisation [58].
- Spatial differences and varying affectedness: Building on the considerations about individual characteristics of citizen scientists, we assume regional differences in the response rate to media coverage [45]. On the one hand, these regional variations in media coverage may be due to the respective different composition and presence of local newspapers, television and radio stations [59]. Regions may also differ by levels of mosquito occurrence and nuisance. Thus, in regions with higher levels of mosquito affectedness, a higher response rate may occur.
- Media landscape: With regard to the media landscape, there are different types of media (e.g. broadcast, print, online) with different reaches (regional versus national) or target groups (tabloid versus high quality journalism). They therefore filter information about the project and put a message into different contexts, which influences the efficacy of a message in different ways [60, 61]. We assume that different media types may have different effects on the response rate of citizen scientists to the ‘Mückenatlas’ project as indicated by previous studies [41, 43].

The aim of this study is to gain insights on the connection between media coverage and the activation of citizens to participate in citizen science projects. We draw on aggregations of a media monitoring service between 2014 and 2017 to test our hypotheses built on the five influencing factors stated above. Based on real data, we explored how effective the ‘Mückenatlas’ mass media approach turned out and thus provide insights for the targeted design of communication strategies for other citizen science projects.

## 3. Material and methods

The study is exploratory and based on data that was not originally collected for the purposes of this study, but to check the effectiveness of the public relations strategy in the context of corporate communications. This is why the dataset has some deficiencies, such as missing distribution data, limited information about the reach or impact area of publishers or outdated links to online media reports. In addition, the media type ‘print’ is only present in 2017, while the media type ‘online’ is lacking for this year. Nevertheless, the data present a rare opportunity to look for initial clues about the relationship between media coverage and participation.

### 3.1 Media dataset

The raw data of media reports originated from Argus Data Insights, a media monitoring service that provides information on the media presence of companies, also called media clipping. The company was assigned from 2014 until the end of 2017 by the ZALF to find and consolidate media content for monthly reporting. The service’s engine searched the media types ‘print’, ‘radio’, ‘television’, ‘online’ and ‘news agencies’ (dpa) on a national level by using the keywords ‘Mückenatlas’, alone or in combination with ‘Leibniz-Zentrum für Agrarlandschaftsforschung’. Due to the exit of Argus Data Insights from the online business in 2017, the composition of ‘television’, ‘radio’, ‘print’ and ‘online’ varies greatly from year to year, so any temporal evaluation of media types would be meaningless.

The media clipping dataset contained 1072 observations of media reports from 2014 to 2017, with 9 automatically generated covariates, such as date, title and media type (see S1 Table in S2 File). To prevent duplication in the descriptive analysis, observations from dpa were not considered, resulting in a total of 934 media reports for quantitative analysis. We excluded dpa-releases, because these are editorial contents offered to media, companies and organisations, i.e. they are inaccessible for the public and not published directly, but are picked up by local, regional or national media houses which in most cases also take over the entire wording of the original release. dpa-releases containing the ’Mückenatlas’ are usually created via the regional studio Berlin-Brandenburg and are then taken over by other dpa offices (at the national or the federal state level) and offered to the media of their respective impact area.

To approximate the spatial distribution of media reports, we created the variables ‘municipality’ (the smallest administrative division in Germany), ‘xvalue’ and ‘yvalue’ for geo-references and ‘reach’. For the ‘municipality’ variable, media reports that originated from a certain municipality and targeted a regional audience were assigned the exact municipality name. In all other cases, the media reports were categorised as ‘federal’ or ‘national’ depending on their reach. For those media reports where a specific municipality could be specified, the corresponding geo-coordinates of the community centre were recorded in ‘xvalue’ and ‘yvalue’. For the variable ‘reach’, entries with a geo-reference were assigned to ‘regional’. Media reports that targeted an audience of one or more federal states were categorised as ‘single federal’ or ‘cross federal’, respectively. For entries with national reach, the categories for ‘reach’ were set to ‘national’ (see S2 Table in S2 File for method description, examples and complete list).

### 3.2 ‘Mückenatlas’ dataset

Data of mosquito submissions to the ‘Mückenatlas’ were extracted from CULBASE on July 31^st^, 2018. According to the time period covered by the media clipping dataset, we selected mosquito collections of the years 2014 to 2017 and used the provided geo-references for locating the samples. Each entry of the dataset represents the report of one mosquito species from one location (catching site) independently of specimen counts per sample site, hereafter referred to as ‘submission’. The resulting dataset comprised 16,610 submissions.

### 3.3 Statistical analysis

#### 3.3.1. Project awareness and knowledge (i)

To investigate our hypothesis of a positive correlation between the number of media reports and the number of submissions, we first examined the temporal relationship. The monthly frequencies of both datasets were plotted across all years and tested for a possible time lag between the maxima of media reports and submission numbers using Spearman cross-correlation analysis.

To examine a possible spatial association between number of media reports and number of submissions, we applied a spatial pattern analysis with two spatial point datasets. The first dataset consisted of the geo-locations of ‘Mückenatlas’ submissions. The second dataset contained those media reports for which we were able to verify a municipality and to which a geo-location could thus be assigned. Consequently, media reports with federal or national reach were excluded from this analysis.

To visually inspect the geographical distribution of media reports and submissions across Germany, we produced density plots, which suggested spatial clustering for both submissions and media reports. To assess the level of clustering of both point pattern datasets, each was individually tested by comparing the geographical distribution of the points to a theoretical point pattern of complete spatial randomness, using the Kolmogorov-Smirnov test. These initial assessments led us to assume that the two point patterns are similarly clustered throughout the country, indicating that locations of clusters of media reports are in the same geographical locations as clusters of submissions.

To investigate this potential relative clustering, the bivariate version of Ripley’s K-function (Cross K-function, command *Kcross()* in the R package *spatstat(*)) was used, where *j* is the number of submissions within a certain distance of a media report *i*. A Monte Carlo simulation was applied to statistically test the distinction between the observed Cross K-function and a Cross K-function produced by random labelling, that is, all observations were labelled either as *i* = ‘media report’ or *j* = ‘submission’ while keeping the observed proportions (permutations = 100).

#### 3.3.2. Attention economy (ii) and individual characteristics of citizen scientists and targeted communication (iii)

To identify possible factors influencing the attention and differences in participants’ response rates, we followed three steps. First, we identified exceptional spikes in submission and media report numbers on a temporal scale by a descriptive analysis. Second, we related these findings to the proportion of geographical ‘reach’ categories. Third, we investigated the quality of headline and text of the dpa-release on June 6, 2016, that resulted in a maximum number of following media reports and submissions in comparison to the titles of all other dpa-releases from the dpa regional office of Berlin/Brandenburg. We paid attention to the language style as well as to the contextualisation, especially whether the latter is indicative of a specific event that could have prepared the ground for increased attention to mosquitoes.

#### 3.3.3. Spatial differences and varying affectedness (iv)

We also investigated if differences in the response to media coverage on the level of federal states can be attributed to differences in the media coverage itself or to the degree to which people are affected by mosquitoes. We plotted numbers of submissions and media reports per federal state to identify possible contradictions. In an exemplary fashion, two contradictory federal states with high numbers of submissions but few media reports, and vice versa, were examined for whether there is a significant, quantitative difference in the reach of the respective media reports or in the media types, applying a Chi-square test of homogeneity. A qualitative evaluation of the media contributions per federal state, i.e. a text analysis, could not be carried out because most of the media reports were no longer accessible and therefore no statistically justifiable number of articles was available.

#### 3.3.4. Media landscape (v)

To test whether media types may have different effects on the response rate of citizen scientists to the ‘Mückenatlas’, we analysed the submission forms filled by the participants and contrasted the results with the proportions of media types from the media clipping dataset. Each submission to the ‘Mückenatlas’ is required to be accompanied by a form filled out by the participant on which contact and collection details are provided: name, address, email or phone number, collection date and site (if different from home address) and free remarks for collection-site description. In addition, an open question is raised on the submission form as to how the participants became aware of the ‘Mückenatlas’, with the examples ‘internet’, ‘radio’, ‘acquaintances’ and ‘television’ provided as possible answers (S1 File). We selected 2098 submission forms of the year 2017 (approximately 10% of total submission numbers at the date of data extraction) with respect to the open question, as that year represents the most recent year of overlap with available data on media reports. Of these 2098 forms, 25.1% (n = 526) contained no response and were removed from the analysis. Of the remaining 1572 forms, 78 were considered invalid due to non-interpretable descriptions. To analyse the valid answers to the open question, they were manually binned to ‘newspaper’, ‘magazine’, ‘television’, ‘radio’, ‘internet’, ‘personal communication’ or ‘other’. As the categorisation was conducted by one and the same person from the authors’ collective, we waived the inter-observer reliability check.

All analyses were carried out with R, version 3.6.3 [62]. Datasets and plot figures were created using the R packages *ggplot2* [63], *tidyr* [64], *dplyr* [65], *scales* [66] and *zoo* [67]. Statistics were conducted with packages *rstatix* [68] and *tseries* [69]. For point pattern analysis, we deployed the packages *rgdal* [70], *raster* [71], *spatstat* [72] and *maptools* [73], while the colour scheme was created with *viridis* [74].

### 3.4 Ethical approval

Insect samples were provided voluntarily by citizen scientists. By signing the submission form that includes comprehensive information on data privacy, participants agreed with the processing of their personal data according to EU General Data Protection Regulation. For the storage and analysis of the mosquito data, the personal data of the participants (i.e. the contact information for each response) is separated from the information of the submitted samples, with the exception of the geo-reference of the catch locations, thereby further anonymising the collected data. These anonymised submissions were also used for this study outside the actual research purpose of the ‘Mückenatlas’ in order to protect the personal rights of the citizen scientists.

## 4. Results

### 4.1 Project awareness and knowledge (i)

**Fig 1:**
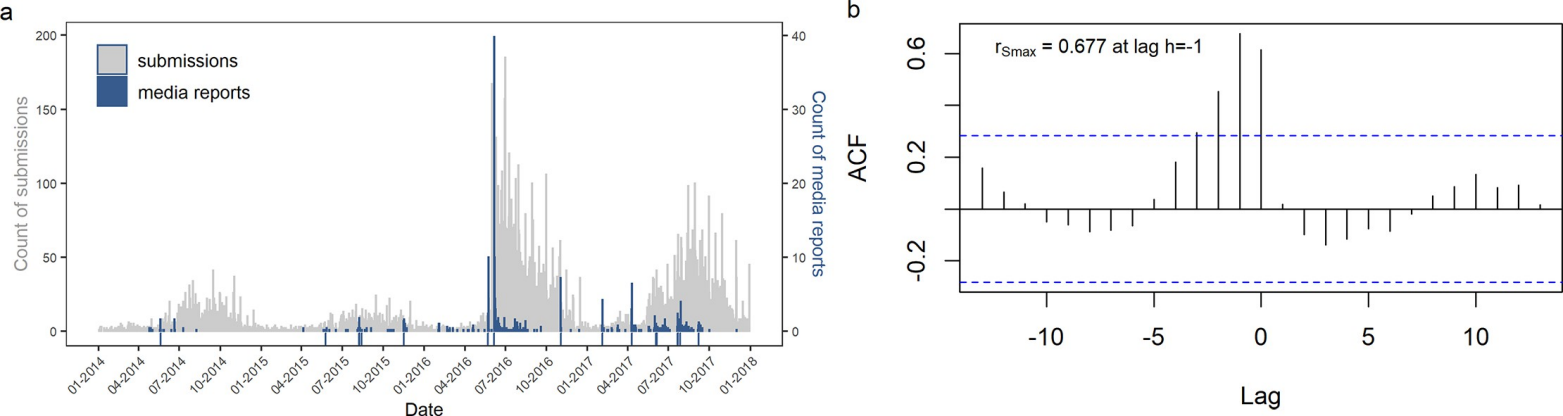
Temporal correlation of submissions of media reports between 2014 and 2017. (a) Combined plot of numbers of media reports (right y-axis) and submissions (left y-axis). The blue coloured ticks on the x-axis represent the dates of releases by the dpa regional studio Berlin-Brandenburg. Note the media echo and submission response to the dpa-release on June 6, 2016. (b) Cross-correlation analysis shows the highest value at -1, the blue dashed lines indicate a confidence threshold for α = 0.05.

A comparison of the monthly numbers of submissions and of media reports over the considered time period (Fig 1A) shows that most peaks in media reports are concurrent with or followed by peaks in submissions. Fig 1B implies a time lag between peaks in media reports and submissions. Spearman cross-correlation analysis showed that the maximum correlation of r_s_ = 0.677 (p < 0.001) is at a value of h = -1, which means that most submissions occur about one month after a maximum of media coverage (Fig 1B).

**Fig 2:**
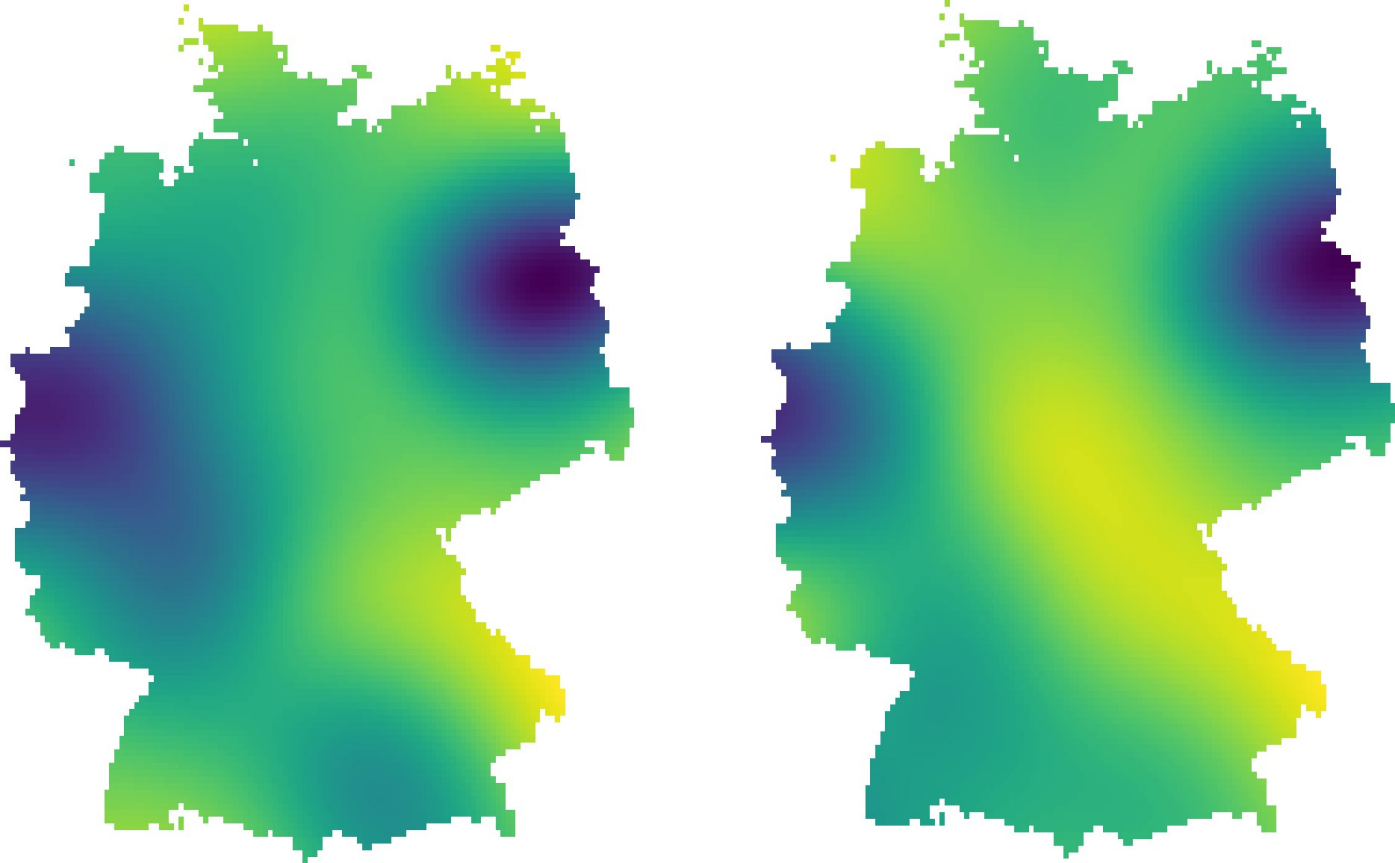
Density plots with gradients of spatial aggregation of media reports (left) and ‘Mückenatlas’ submissions (right). The darker the colour, the more submissions or media reports, respectively.

The density plots show spatial aggregations of both ‘Mückenatlas’ submissions and media reports (Fig 2). Testing for complete spatial randomness of the individual datasets indicates that both point patterns are not randomly distributed, as for both comparisons of observed vs expected (theoretical homogenous Poisson process) patterns, cumulative distributions are significantly different (both p < 0.01). Both point datasets are clustered, as each observed curve lies above the theoretical Poisson process curve, and therefore empirical values are greater than theoretical values (S2 Fig in S2 File).

Bivariate Ripley’s K function output indicates that the locations of submissions are closer to the locations of media reports than complete spatial randomness would expect (S3 Fig in S2 File). A Monte Carlo simulation with 100 permutations to test whether observed and theoretical mean curves were significantly different was run for distances up to 80,000 m. This procedure calculated an upper and lower simulation envelope for random labelling at a 99.98% significance level. The result implies attraction, indicating that the locations of media reports and ‘Mückenatlas’ submissions are closer together than would be expected under random labelling (S3 Fig in S2 File).

### 4.2 Attention economy (ii) and individual characteristics of citizen scientists and targeted communication (iii)

**Fig 3:**
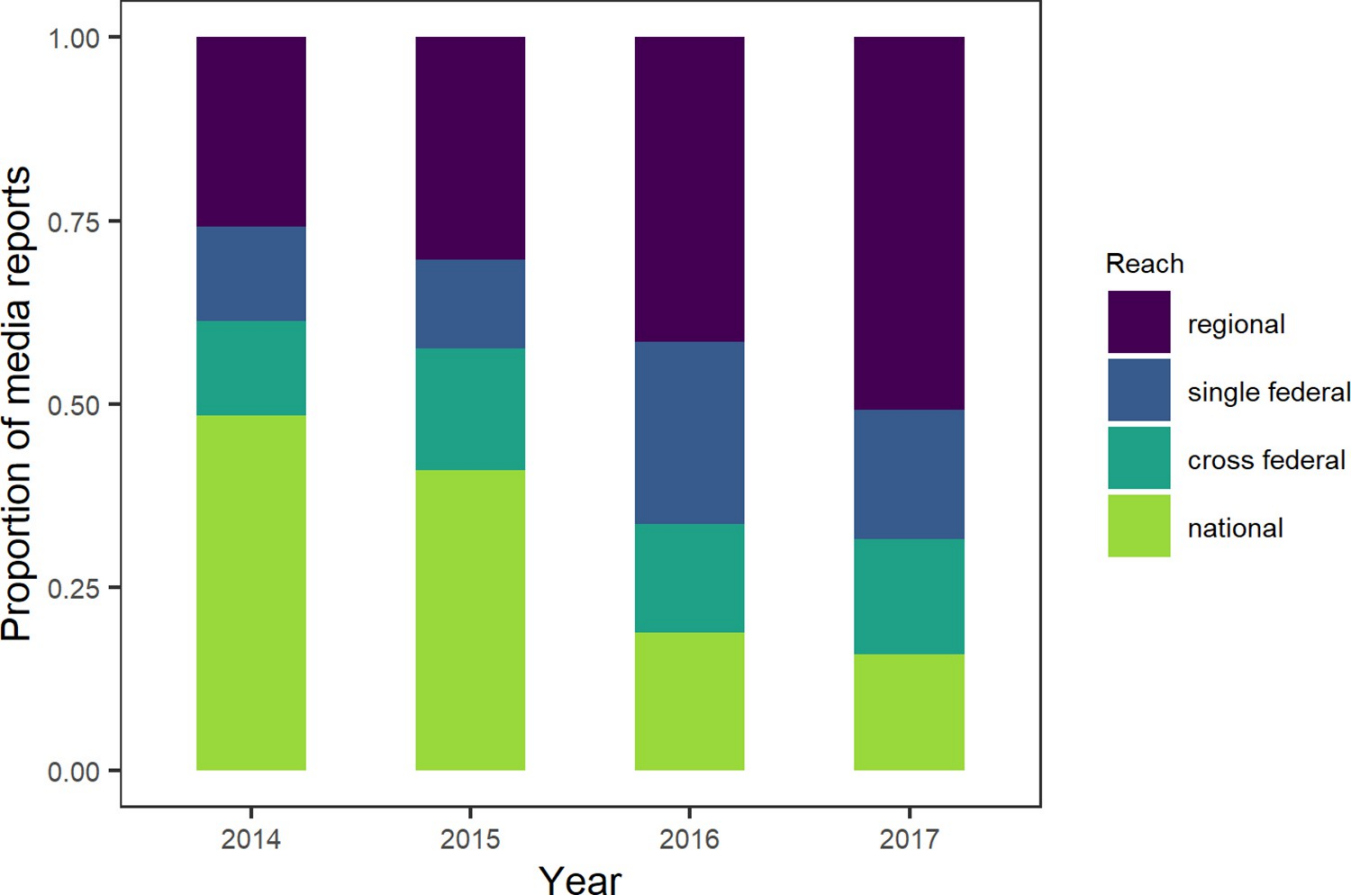
Annual proportions of geographical reach of media reports. While media reports with national reach predominated in 2014/2015, the share of regional and single federal category massively increased in 2016/2017.

Both patterns in Fig 1A display more media reports and submissions during the main mosquito season from May to September, compared to the autumn and winter months. Strikingly, over 50% of the reports were published in 2016 (n = 542), with an outstanding majority in June 2016 (n = 350, 37.5% of total reports). This suggests that an external factor positively affected the activation of participants. In 2017, submission and media report frequency kept to be high compared to 2014 and 2015, but not reaching the levels of 2016 (see S3 Table in S2 File for annual numbers). A similar pattern can be seen in Fig 3: The distributions of geographical reach of media reports are more alike for 2014/2015 and 2016/2017. For the latter years, the amount of media reports with regional and single federal reach predominates.

The most successful dpa-release happened on June 6, 2016, and resulted in 199 media reports and a subsequent maximum in submissions on that very day (Fig 1A). Therefore, we picked out this day to look at the headlines of the dpa-release as well as the media reports on that day and compared them to the headlines of all other dpa-releases. Most reports on that day had the same title as the original dpa-release:

*Forscher-Bitte: Bürger sollen Mücken schicken / Researcher’s plea: Citizens should send mosquitoes*

Also, some variations were used, but these all contained a concrete call-to-action similar to the dpa-release headline, such as:

*Forscher: Schickt uns Mücken! / Researchers: Send us mosquitoes!*

*Bitte von Forschern: Mücken einfangen und einsenden / Request from researchers: Capture and submit mosquitoes*

*Bürger sollen Mücken fangen und einschicken / Citizens should catch and submit mosquitoes*

*Forscher: Schickt uns Mücken! / Researchers: Send us mosquitoes!*

Opposed to that, dpa-release headlines outside that date did not appeal to the citizens for support (see S4 Table in S2 File) in such a direct way:

*Auch Mücken und Nacktschnecken lieben den Start-up-Sommer / Mosquitoes and slugs also love the start-up summer*

*Mückenatlas: Forscher fahnden nach neuen Stechmückenarten / ‘Mückenatlas’: Researchers search for new mosquito species*

*Biologin: Mückensaison bislang lau / Biologist: Mosquito season so far meagre*

*Jede Mücke zählt—Wie sich Exoten in Deutschland etablieren / Every mosquito counts—How exotics establish in Germany*

*Sommer, Sonne, Mücke—Plagegeister surren wieder / Summer, sun, mosquitoes—pests are buzzing again*

*Mückenjäger fangen 30 000 Tiere für die Forschung / Mosquito hunters catch 30,000 animals for research*

By retrieving still existing online articles based on the dpa-release of June 6, 2016, and looking at the content, we found additional information why this release was followed by a high media echo and many submissions. An included quote from the project leader adopted by the media substantiates the call-to-action and places it in a timely context, namely with regard to the Zika virus epidemic that occurred in South America in 2016:

*«Durch die in Europa in den letzten Jahren zunehmenden Ausbrüche von Stechmücken-übertragenen Krankheiten wie Dengue-, Westnil- oder Chikungunya-Fieber sowie den jüngsten Zika-Virus-Ausbruch in Südamerika wurde die aktuelle Bedeutung von Stechmücken als Krankheitsüberträger unter Beweis gestellt», erklärte Walther. «Zur Risikoabschätzung benötigen wir dringend Daten zur Verbreitung der in Deutschland vorkommenden invasiven und einheimischen Arten.» /*

*"The increasing outbreaks of mosquito-borne diseases such as dengue, West Nile and chikungunya fever in Europe in recent years, as well as the recent Zika virus outbreak in South America, have demonstrated the current importance of mosquitoes as vectors of disease agents," Walther explained. "To assess the risk, we urgently need data on the distribution of invasive and native species in Germany*.

### 4.3 Spatial differences and varying affectedness (iv)

**Fig 4:**
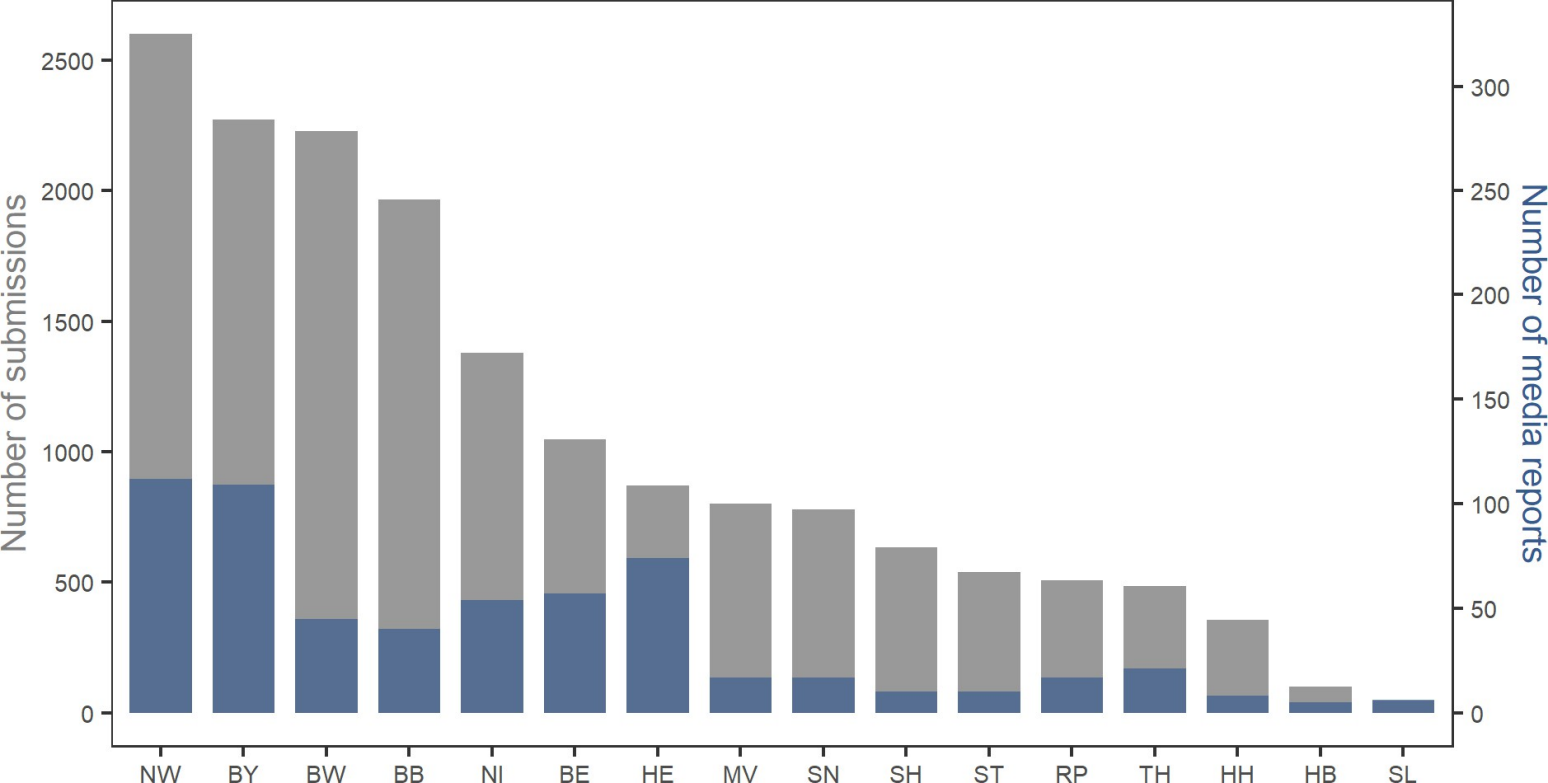
Numbers of submission (left y-axis) and media reports (right y-axis) by federal state. (NW = North Rhine-Westphalia, BY = Bavaria, BW = Baden-Wuerttemberg, BB = Brandenburg, NS = Lower Saxony, BE = Berlin, HE = Hesse, MV = Mecklenburg-Western Pomerania, SN = Saxony, SH = Schleswig-Holstein, ST = Saxony-Anhalt, RP = Rhineland-Palatinate, TH = Thuringia, HH = Hamburg, HB = Bremen, SL = Saarland). Spearman’s correlation coefficient shows a highly positive correlation (r_s_ = 0.878, p < 0.001).

In terms of spatial association of submissions and media report numbers aggregated by federal state, we found contradictory results. Most submissions do not necessarily come from federal states with high media coverage, e.g. Berlin (BE) or Hesse (HE), and a small number of media reports can as well result in high submission numbers, as observed in Baden-Wuerttemberg (BW) and Brandenburg (BB) (Fig 4).

Focusing at the federal states of Baden-Wuerttemberg and Hesse as examples for reversed characteristics, the omnibus Chi-square test revealed no significant differences in the number of media types (*χ2* (1, n = 119) = 2.13, p > 0.05) or reach of media reports (*χ2* (1, n = 119) = 0.253, p > 0.05) (Table 1). Differences in participant responses must be traced back to either the quality of the media reports or the affectedness by mosquitoes.

**Table 1:** Number of media reports and outcome of chi-square test of homogeneity.

	**Media type**					
**Federal state**	*online*	*print*	*radio*	*television*	*χ2*	*p-value*
Baden-Wuerttemberg	26 (57.78)	7 (15.56)	7 (15.56)	5 (11.11)	2.13	0.545
Hesse	50 (67.57)	8 (10.81)	12 (16.22)	4 (5.41)		
	**Reach**					
**Federal state**	*Single federal*		*regional*		*χ2*	*p-value*
Baden-Wuerttemberg	32 (71.7)		13 (28.9)		0.253	0.615
Hesse	48 (64.9)		26 (35.1)			

Carried out for media type and geographical reach for the federal states of Baden-Wuerttemberg and Hesse. Integers are absolute numbers of media reports, percentages are provided in brackets.

### 4.4 Media landscape (v)

**Fig 5:**
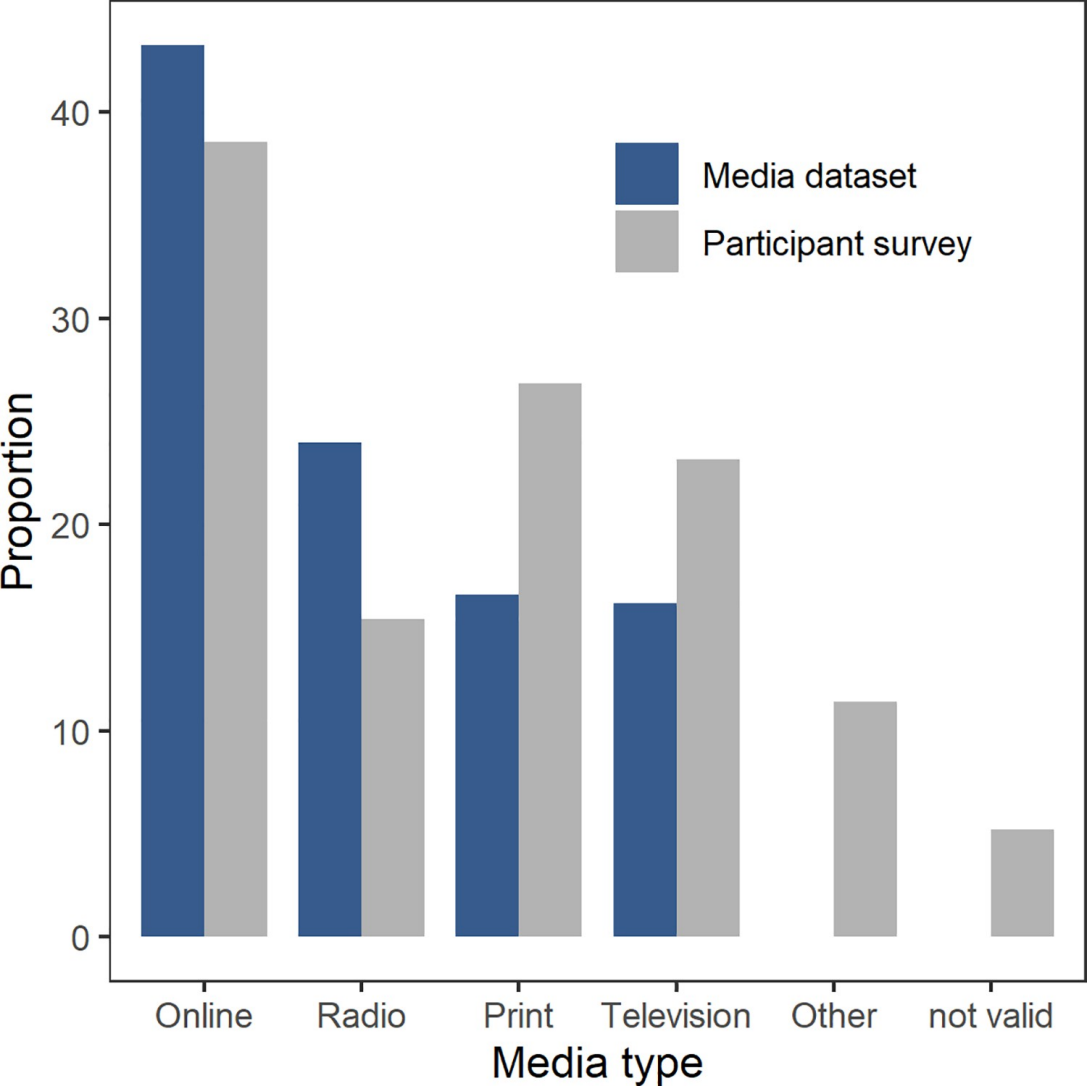
Comparison of the proportions of media types from the media clipping dataset and those from the participant responses. For the participant responses, the category ‘print’ also includes ‘magazines’; the ‘personal’ category was assigned to ‘other’.

According to the media clipping dataset about the ‘Mückenatlas’ from 2014 to 2017, most media reports were published online (n = 406, 43.3%), followed by radio broadcasts (n = 224, 24.0%), printed articles (n = 155, 16.6%) and television programmes (n = 152, 16.2%). By comparison, the evaluation of the submission forms showed that most participants became aware of the citizen science project ‘Mückenatlas’ online, followed by television, newspaper, radio, magazines, personal communication and other sources (e.g. events, invited lectures at community colleges or health departments) (Table 2, Fig 5).

**Table 2:** Binned answers of the free-form response to the question ‘where did you hear about the ‘Mückenatlas’?’.

Source	online	television	newspaper	radio	magazine	personal	other
N %	576 38.6	346 23.2	255 17.1	230 15.4	146 9.8	129 8.6	41 2.7

The percentages refer to the number of submission forms including invalid answers (n = 1494). The number of individual answers exceeds the number of valid submission forms, since 11.9% (n = 205) of the participants named more than one source of information.

## 5. Discussion

The ‘Mückenatlas’ is both a science communication and a research tool: information about mosquitoes is distributed to a broad public, and at the same time large amounts of data are collected for research. As participation does not require any skills or training–except for catching a mosquito without smashing it–the project counts on submissions from people who heard about the ‘Mückenatlas’ and rather spontaneously decide to take part. Therefore, we use mass media to reach the largest possible share of the population, because the more new participants submit mosquitoes, the more detailed the map of collections and the better the geographical coverage of Germany becomes. By investigating five influencing factors for the responsiveness to mass media communication events, we estimated how effective the ‘Mückenatlas’ mass media strategy actually is to reach the project’s scientific goal.

### 5.1 Number of media reports impact submission frequency on a spatio-temporal scale (i)

Our hypothesis of a positive correlation between the number of media reports and the number of submissions was confirmed for both a temporal and spatial scale. Temporal associations between media campaigns and participants’ activation have been attested before, e.g. a social media campaign and press release led to increased download and subsequent usage of an app for water body observation [43].

The time lag of around one month between the peak in media report and mosquito submissions could be an adaptive form of a typical response to citizens’ exposure of a topic via mass media called *short-term fluctuation change* [61]: the media report induces a peak in knowledge and behavioural change that subsides after a couple of days. In case of the ‘Mückenatlas’, this time span might be extended a bit, as a mosquito is not always immediately available for catching but might still trigger the memory on the project a few weeks later.

The spatial analysis revealed that both point datasets are spatially clustered, with two hotspots of different sizes each in North Rhine-Westphalia (West Germany) and Brandenburg/Berlin (North-East Germany). Although the spatial distributions of the reports and submissions are not exactly the same, they are similar and locations of submissions are closer to the locations of media reports than would be expected by chance. Even if this connection seems logical, it has not yet been demonstrated in the context of citizen science. This finding highlights the potential of using the media to attract more records from underrepresented regions or areas of special interest through targeted communication of large-scale citizen science projects, such as approaching regional press or customising participation campaigns for local communities [40, 75].

### 5.2 Heightened attention to mosquitoes and a direct call for support maximises participation (ii-iii)

In addition to the positive correlation of numbers of media reports and submissions, Fig 1A shows repetitive temporal patterns and exceptional events. Both media reports and submissions underlie the seasonality of mosquito occurrence: the more mosquitoes there are, the higher the probability of someone participating and–presumably–the more they become an issue worth reporting. The fact that citizen observations and media reports reflect the phenology of the study object is a well-known phenomenon in citizen science projects for monitoring biodiversity [44, 76, 77]. Mosquitoes also make a popular topic in the summer slump, when politicians withdraw for vacation and themes to report on tend to be scarce.

In general, Fig 1A also displays more media reports in 2016 and 2017 than in the previous years, which was mainly due to an increase in regional media reports (Fig 2) and is reflected by a high level in numbers of submissions throughout these years. The phenomenon emanates from a peak of media reports in June 2016 and could be explained by various factors.

First, the news [78] that the Zika virus is transmitted by mosquitoes and leads to brain damage in newborns if the mother has become infected during pregnancy had been picked up and spread by the German media in early 2016, thereby paving the way for greater attention to the relevance of mosquitoes for public health. Hence, in the context of the attention economy concept, the most successful dpa-release on June 6, 2016, had already the advantage of a heightened awareness to the topic.

Second, the style of the title of this dpa-release and those of the following media reports for which title information was available, was very distinct to that of all other dpa-releases by a concrete call to the public to submit mosquitoes. In addition, the text of the dpa-release, which was probably copied by many of the following media articles, included a quote by the project leader that related the importance of participation directly to the Zika virus. Chu et al. [40] reported a similar experience with a 2010 press release that combined a call for support and a researcher’s quote about the scientific importance of people’s participation in the citizen science project NestWatch. Moreover, health-related issues in connection with citizen science seem to be a very interesting topic for journalists in general [44]. In contrast, other dpa-release titles referred to a possible upcoming of severe mosquito plagues, how mosquitoes may react to predominating weather conditions or in some cases to the invasion progress of alien species (S4 Table in S2 File).

This triad of existing media attention towards mosquitoes, a direct appeal to citizens and relevance to health may have led to the maximum of submissions in June 2016. Continued strong coverage likely sustained public attention throughout 2016 and 2017, resulting in still high numbers of mosquito submissions. We assume that, additionally to a wider information reach also through regional media (Fig 2), the public’s concern toward the Zika virus stayed high, which led citizens to use the ‘Mückenatlas’ as a tool of self-care, reassurance and as a communication channel to a trusted authority.

### 5.3 Many factors are responsible for regional differences in submissions (iv)

As assumed, regional differences in the response rate to media coverage could be identified. In accordance of the findings on the spatial correlation of the numbers of submissions with media reports, they also correlate positively on the federal state level. Despite this general correlation, the results are somewhat contradictory when having a closer look (Fig 4). For example, we picked out the federal states of Baden-Wuerttemberg and Hesse, with an inverse number of media reports and submissions, in order to find out whether this contradiction could be caused either by distinct proportions of media types/reach in each case or by differences in mosquito affectedness. Baden-Wuerttemberg is characterised by high submission numbers and low media reports, whereas the opposite is true for Hesse. Since there are no significant differences between media type and reach between the two federal states (Table 2), other factors might cause the contradiction.

Over a length of 420 km, the river Rhine borders Baden-Wuerttemberg with a multitude of riparian and floodplain areas and oxbow lakes. In addition, there are 155 km of shore area of Lake Constance. Warm and humid conditions and fluctuating water levels lead to regular mass developments of mosquitoes in these regions [79], which have been controlled by community organisations since 1910 [80]. In addition to this burden of mosquito nuisance on the human population, the invasive Asian tiger (*Aedes albopictus*) and Asian bush (*Aedes japonicus)* mosquitoes were detected in Baden-Wuerttemberg already in 2007 and 2008, respectively [81, 82]. By contrast, only 107 kilometres of Rhine with major breeding sites for mosquitoes is located in Hesse, where the first invasive mosquito species (*Ae*. *japonicus*) was only recorded in 2015 [36].

A reduced quality of life for many citizens due to nuisance, a long tradition of countermeasures and prolonged media exposure of the impacts of invasive species may therefore have led to a higher affectedness by mosquitoes in the Baden-Wuerttemberg population. Hence, the willingness to participate in mosquito-related initiatives such as the ‘Mückenatlas’ might already have been on a relatively high level there and less dependent on media coverage. However, since 2017 a population of the invasive species *Aedes koreicus* has been occurring in Wiesbaden, Hesse, as the only population in Germany [83]. Whether the accompanying media coverage has a direct impact on the number of submissions from this federal state should become an interesting study object in the coming years.

Another interesting pattern was that, similarly to Baden-Wuerttemberg, the federal states of Mecklenburg-Western Pomerania (MV) and Brandenburg (BB) showed high submission frequencies despite low numbers of media reports (Fig 4, see also [84]). This pattern resulted from a *headquarter effect*, meaning that local communities tend to support their local projects, also known as place-based effects [85].

### 5.4 Different media types have different effects on citizen responsiveness (v)

More than 85% of all participants whose responses were analysed learnt about the ‘Mückenatlas’ through a media channel, thereby highlighting the effectiveness of the project’s communication strategy, as millions of people are reached via mass media per year [44].

However, there are disparities between the proportions of media types that reported on the ‘Mückenatlas’ and the channels through which participants learned about the project. Print media and television are only moderately used to report on the ‘Mückenatlas’, but obviously have an activating effect. Typical television broadcasts that feature the citizen science project are science-related programmes and regional news formats. Print media is still an important source of news on the regional level [86, 87], and the probability that local newspapers report on the ‘Mückenatlas’, e.g. in the context of a local nuisance or an invasion incidence [76], is higher than with national print media.

Although online media seem to be the most effective way of raising awareness of the ‘Mückenatlas’, our results can barely be interpreted due to the lack of comparability of the participants’ response and the media clipping dataset. The ‘online’ category of the media clipping dataset comprises everything from internet-representations of print, television and radio media to pure e-zines to forums. On the other hand, what is behind the ‘online’ category based on the participant answers remains in the dark for the time being, as only a few of them provided more information than ‘the internet’. This is probably also due to the fact that ‘internet’ is listed as an answer example in the question on the submission form, and thus had a suggestive effect. In addition, this category probably covers much more than that of the media clipping dataset, because the ‘Mückenatlas’ homepage, social media or e-mails could also be meant. Radio has the least impact on potential participants, as it appeals to only one sense and is consumed more fluctuating, but also more in passing. Although we cannot derive any evidence-based explanations for these disparities, the results confirm the findings of other studies that different types of media have different effects on participation [23, 41, 43].

The fact that word-of-mouth propaganda (‘personal’), although minor (7.5%) and not supported by marketing, still takes place shows parallels to recruitment processes known from the volunteering literature [88]. Face-to-face communication is another important factor to recruit participants, e.g. at events, especially to reach a more diverse group of participants [89].

### 5.5 Limitations

There are some shortcomings to this study, mainly based on the media clipping dataset. First, there is a lack of online data for 2017, as the institute changed the clipping service for online media. We decided against including a separate dataset from the new service, as it is based on different search algorithms and search terms, making comparisons unfeasible. In addition, print media data is missing for 2014 to 2016, which could skew the dataset towards an overrepresentation of regional reach in 2017.

The spatial distribution of submissions is biased by human population density [84]. However, the number of media houses, and therefore of newspapers, broadcasters and websites, underlie the same effect: in general there are more media in more densely populated areas.

The qualitative title and text analysis is based on dpa-releases, as these trigger the majority of media reports with mostly the same or very similar wording. However, there are also a considerable number of media reports that are independent of these dpa-releases. This information was excluded in the qualitative analysis. In order to make a more reliable statement on spatial and temporal effectiveness in connection with text quality, the inclusion and retrieval of all articles would be necessary. Future studies could use experimental approaches with differently designed press or dpa-releases to test our findings and hypotheses.

## 6. Conclusions and recommendations

The clear positive correlation between numbers of media reports and numbers of mosquito submissions demonstrate that communication of the project via the mass media as multipliers is important to activate participants. However, the resulting temporal and spatial clustering of the submissions is also a potential source of bias in opportunistically collected data. This has been observed in other studies [90, 91], but has never been investigated specifically in the context of citizen science. We suggest that data bias induced by media coverage should be further explored in the future.

In times when public attention is a scarce commodity, the title of a media report can be a decisive hook. With a targeted call-to-action in the title, we achieved maximum participation and, rather unintentionally, took advantage of increased media and public attention due to a current human health risk situation caused by mosquitoes. Since other projects have also made this experience [40], it could be a good indication how to design catchy titles for press releases or newsletters. In addition, issue management in the sense of horizon scanning is recommended to optimise the timing of communication activities [44].

Despite the clear spatial association between media reports and submissions on a national level, differences in regional participation frequency are presumably due to multiple factors. For mosquitoes, we could demonstrate that human affectedness might play an important role. We commend to use the level of affectedness towards the subject of interest in a more targeted way, e.g. appeal to pet owners to collect ticks or to hobby gardeners to monitor invasive environmental weeds.

We advise to involve the media and establish reliable relations with press representatives already when planning new or reinvigorate existing citizen science projects [92]. For example, the ZALF ‘Mückenatlas’ project lead maintains good connections to the regional dpa-office representing Berlin and Brandenburg. The co-operation works mutually: we can approach them, e.g. when submission frequency is low, and they can contact us whenever an expert for mosquitoes is needed. Continuous communication via the media is particularly recommended and important for projects that aim to achieve a large and finely granulated spatial coverage over a considerable period of time in order to continuously attract new participants and to remind citizens who have already been contributing. This is especially true for projects that rely on contributions from ‘dabblers’ [93] and do not require participant training or the provision with certain equipment.

However, the media can only be the spark that triggers pre-existing intrinsic and extrinsic motivational factors of the citizens, which need to be investigated in further studies. Knowing more about the demographic background and motivations of the citizen scientists would allow for customised marketing campaigns that might lead to constantly higher numbers of submissions compared to an undifferentiated ‘scattergun approach’ via the mass media [39].

## Supporting information

S1 File Submission form. This template is available for download at www.mueckenatlas.com and in paper form on request at the project office (German only).
(PDF)

S2 File (PDF)
