# Supplementary material for: How media presence triggers participation in citizen science—The case of the mosquito monitoring project ‘Mückenatlas‘
Source: PLoS One. 2022 Feb 17;17(2):e0262850. doi: 10.1371/journal.pone.0262850 (PMC8853470; doi:10.1371/journal.pone.0262850)
Supplement: S1 File — This template is available for download at www.mueckenatlas.com and in paper form on request at the project office (German only). (PDF) [file pone.0262850.s001.pdf]

Mückenatlas

Einsendeformular

* Pflichtangaben

**Absender**	
**Name***	**Straße, Hausnummer***
	**PLZ, Ort (ggf. Ortsteil)*** Beispiel: 16866 Kyritz oder 16866 Kyritz OT Holzhausen
**E-Mail-Adresse** (bitte geben Sie die E -Mail-Adresse für eine Rückantwort an)	Sollen Angaben zu Ihrer Person bei Ihrem Fundort auf der Karte der Sammler auf www.mueckenatlas.com gemacht werden? Bitte kreuzen Sie an. * **Ja** **Nein** Falls ja, in welcher Form? * (Beispiel: Name, Initialen, Pseudonym)
**Telefonnummer**	
**Wie sind Sie auf den Mückenatlas aufmerksam geworden? (Beispiel: Internet, Funk, Bekannte, Fernsehen o.ä.)**	

**Ort und Zeit des Fundes** * (bitte nur an geben, wenn von oben abweichend)		
**Straße, Hausnummer**	**Geokoordinaten (z.B. von google maps)**	**Fangdatum**
**PLZ, Ort (ggf. Ortsteil)**		
**Weitere Einzelheiten zum Fundort (z.B. Wald, Wiese, Garten, Keller, Wohnzimmer o.ä.)**		

Damit wir Ihre Daten verarbeiten können, erklären Sie sich bitte durch Ihre Unterschrift mit der rückseitigen Datenschutzerklärung einverstanden.

X Datum, Unterschrift

Leibniz-Zentrum für Agrarlandschaftsforschung
„Mückenatlas“
Eberswalder Str. 84m
15374 Müncheberg

Bitte senden Sie Ihre Post an links stehende Adresse:

Datenschutz auf einen Blick

Allgemeine Hinweise
Die folgenden Hinweise geben einen einfachen Überblick darüber, was mit Ihren personenbezogenen Daten passiert, wenn Sie unser Einsendeformular benutzen. Personenbezogene Daten sind alle Daten, mit denen Sie persönlich identifiziert werden können. Ausführliche Informationen zum Thema Datenschutz entnehmen Sie unserer unter diesem Text aufgeführten Datenschutzerklärung.

Datenerfassung durch das Einsendeformular

Wer ist verantwortlich für die Datenerfassung durch das Einsendeformular? Die Datenverarbeitung durch das Einsendeformular erfolgt durch die für den Mückenatlas verantwortliche Arbeitsgruppe. Die Kontaktdaten können Sie dieser Datenschutzerklärung entnehmen.

Wie erfassen wir Ihre Daten? Ihre Daten werden dadurch erhoben, dass Sie uns diese mitteilen.

Wofür nutzen wir Ihre Daten? Ihre Daten werden in eine Datenbank zur Kartierung der Stechmücken in Deutschland eingespeist und zu wissenschaftlichen Zwecken anonymisiert ausgewertet. Falls Sie die entsprechende Option auf dem Einsendeformular gewählt haben, erscheinen Sie neben Ihrem Fangort in der gewählten Form in der Karte der Sammler auf der Webseite www.mueckenatlas.com. Des Weiteren nutzen wir Ihre Daten für eine persönliche Rückantwort mit Angaben zu Ihrem Fang.

Welche Rechte haben Sie bezüglich Ihrer Daten? Sie haben jederzeit das Recht unentgeltlich Auskunft über Herkunft, Empfänger und Zweck Ihrer gespeicherten personenbezogenen Daten zu erhalten. Sie haben außerdem ein Recht, die Berichtigung, Sperrung oder Löschung dieser Daten zu verlangen. Hierzu sowie zu weiteren Fragen zum Thema Datenschutz können Sie sich jederzeit unter der in dieser Datenschutzerklärung angegebenen Adresse an uns wenden. Des Weiteren steht Ihnen ein Beschwerderecht bei der zuständigen Aufsichtsbehörde zu.

Allgemeine Hinweise und Pflichtinformationen

Datenschutz
Das Team vom Mückenatlas nimmt den Schutz Ihrer persönlichen Daten sehr ernst. Wir behandeln Ihre personenbezogenen Daten vertraulich und entsprechend der gesetzlichen Datenschutzvorschriften sowie dieser Datenschutzerklärung. Wenn Sie dieses Formular benutzen, werden verschiedene personenbezogene Daten erhoben. Personenbezogene Daten sind Daten, mit denen Sie persönlich identifiziert werden können. Die vorliegende Datenschutzerklärung erläutert, welche Daten wir erheben und wofür wir sie nutzen. Sie erläutert auch, wie und zu welchem Zweck das geschieht.

Hinweis zur verantwortlichen Stelle
Die verantwortliche Stelle für die Datenverarbeitung durch dieses Einsendeformular ist:

Leibniz-Zentrum für Agrarlandschaftsforschung (ZALF) e.V.
Dr. Doreen Werner
Eberswalder Straße 84
15374 Müncheberg
E-Mail: mueckenatlas@zalf.de

Verantwortliche Stelle ist die natürliche oder juristische Person, die allein oder gemeinsam mit anderen über die Zwecke und Mittel der Verarbeitung von personenbezogenen Daten (z.B. Namen, E-Mail-Adressen o. Ä.) entscheidet.

Widerruf Ihrer Einwilligung zur Datenverarbeitung
Viele Datenverarbeitungsvorgänge sind nur mit Ihrer ausdrücklichen Einwilligung möglich. Sie können eine bereits erteilte Einwilligung jederzeit widerrufen. Dazu reicht eine formlose Mitteilung per E-Mail an uns. Die Rechtmäßigkeit der bis zum Widerruf erfolgten Datenverarbeitung bleibt vom Widerruf unberührt.

Beschwerderecht bei der zuständigen Aufsichtsbehörde
Im Falle datenschutzrechtlicher Verstöße steht dem Betroffenen ein Beschwerderecht bei der zuständigen Aufsichtsbehörde zu. Zuständige Aufsichtsbehörde in datenschutzrechtlichen Fragen ist der Landesdatenschutzbeauftragte des Bundeslandes, in dem unser Unternehmen seinen Sitz hat. Eine Liste der Datenschutzbeauftragten sowie deren Kontaktdaten können folgendem Link entnommen werden: https://www.bfdi.bund.de/DE/Infothek/Anschriften_Links/anschriften_links-node.html.

Recht auf Datenübertragbarkeit
Sie haben das Recht, Daten, die wir auf Grundlage Ihrer Einwilligung oder in Erfüllung eines Vertrags automatisiert verarbeiten, an sich oder an einen Dritten in einem gängigen, maschinenlesbaren Format aushändigen zu lassen. Sofern Sie die direkte Übertragung der Daten an einen anderen Verantwortlichen verlangen, erfolgt dies nur, soweit es technisch machbar ist.

Auskunft, Sperrung, Löschung
Sie haben im Rahmen der geltenden gesetzlichen Bestimmungen jederzeit das Recht auf unentgeltliche Auskunft über Ihre gespeicherten personenbezogenen Daten, deren Herkunft und Empfänger und den Zweck der Datenverarbeitung und ggf. ein Recht auf Berichtigung, Sperrung oder Löschung dieser Daten. Hierzu sowie zu weiteren Fragen zum Thema personenbezogene Daten können Sie sich jederzeit unter der im Impressum angegebenen Adresse an uns wenden.

Datenschutzbeauftragter

Gesetzlich vorgeschriebene Datenschutzbeauftragte

Wir haben für unsere Unternehmen Datenschutzbeauftragte bestellt.:

Leibniz-Zentrum für Agrarlandschaftsforschung (ZALF) e.V.
Dr. Stephan Wirth
Eberswalder Straße 84
15374 Müncheberg
E-Mail: swirth@zalf.de

Friedrich-Loeffler-Institut (FLI)
Martina Rychly
Südufer 10
17493 Greifswald - Insel Riems
E-Mail: Martina.Rychly@fli.de
