# Supplementary material for: How media presence triggers participation in citizen science—The case of the mosquito monitoring project ‘Mückenatlas‘
Source: PLoS One. 2022 Feb 17;17(2):e0262850. doi: 10.1371/journal.pone.0262850 (PMC8853470; doi:10.1371/journal.pone.0262850)
Supplement: S2 File — (PDF) [file pone.0262850.s002.pdf]

S1 Figure

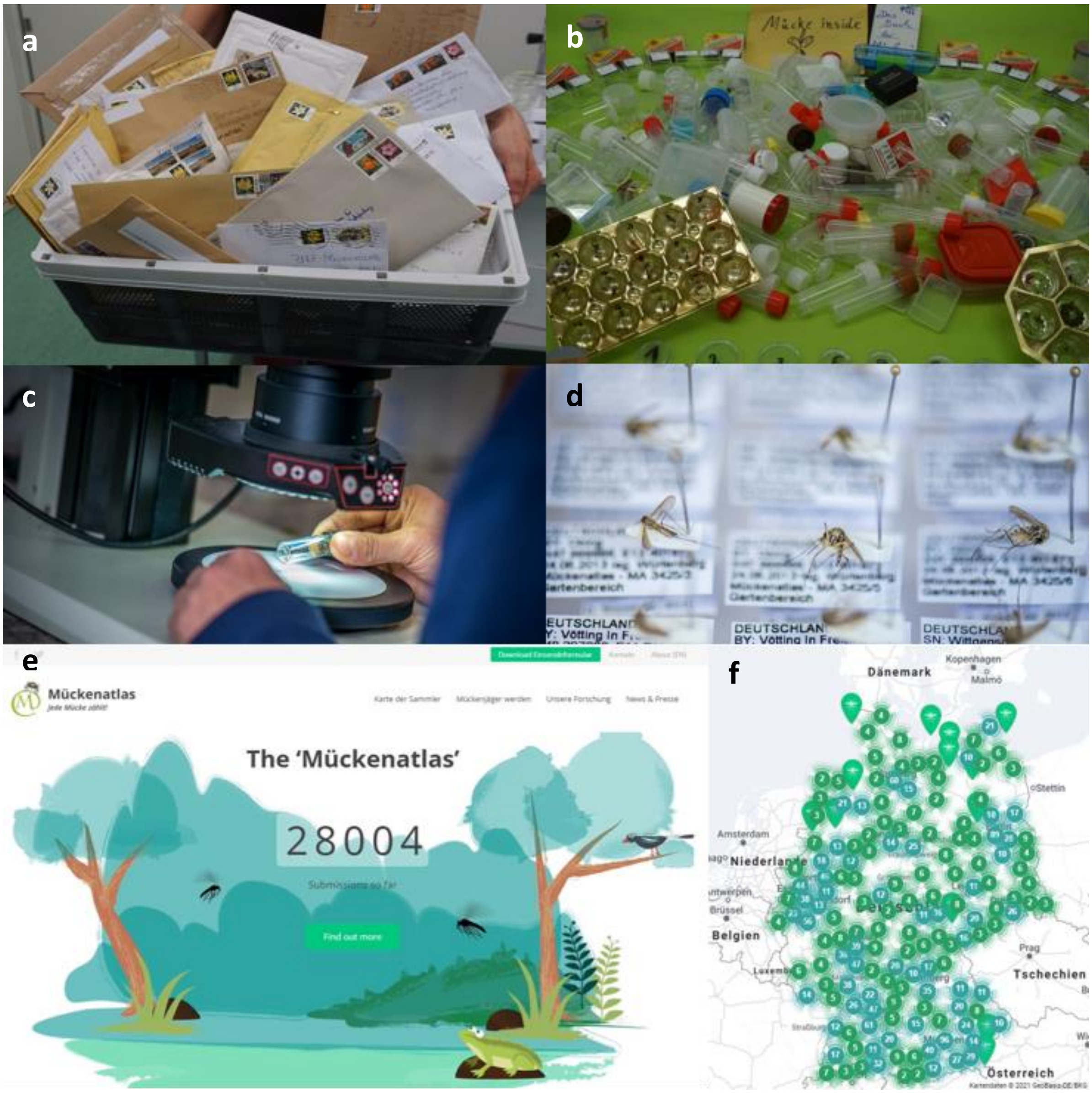

S1 Fig: Impressions from the workflow of the 'Mückenatlas'. (a) During the mosquito season, the submissions arrive in boxes (Photo: Nadja Pernat/ZALF). (b) The participants are very creative in packing the samples (Photo: Nadja Pernat/ZALF). (c) Every mosquito sample is identified to species level and uploaded to the German database for Culicid research (Photo: Jarno Müller/ZALF). (d) Well-preserved and special finds are kept in a reference collection (Photo: Jarno Müller/ZALF). (e) The submission counter on the homepage www.mueckenatlas.com informs about the progress. (f) Every participant who wants to gets a marker with a name or pseudonym on the collector's map on the website.

S2 Figure

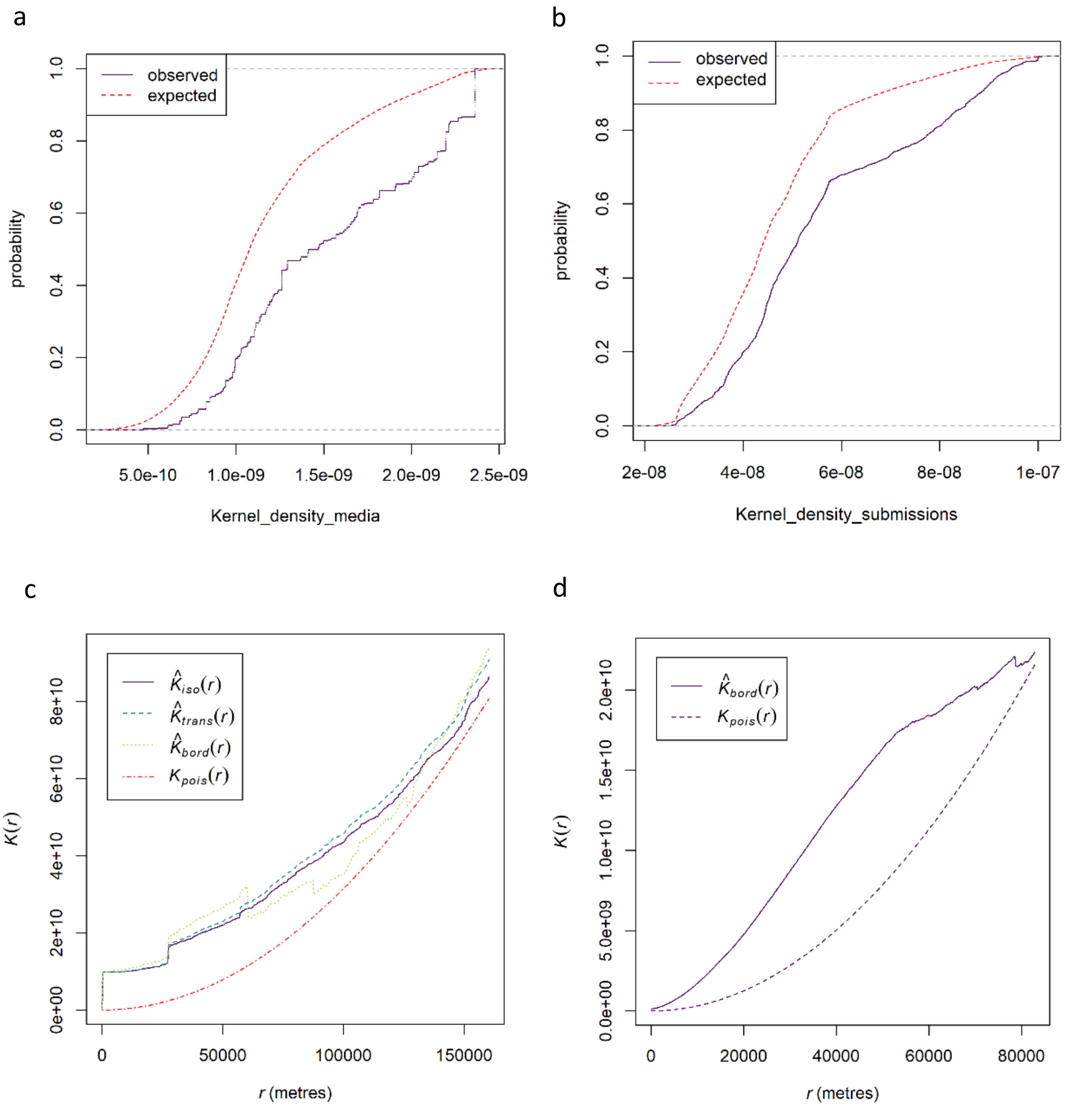

S2 Fig: Plotted results of the Kolgomorov-Smirnov test. Based on Kernel density of geo-references as spatial covariate for (a) media reports and (b) submissions, indicating a non-random distribution of both point pattern datasets across Germany. Ripley's K function suggests a clustering of both point patterns, for (c) media reports and (d) 'Mückenatlas' submissions (right column). As the number of points for the submission dataset exceeds 3000, only border correction estimations (no edge effects) could be calculated for (d).

S3 Figure

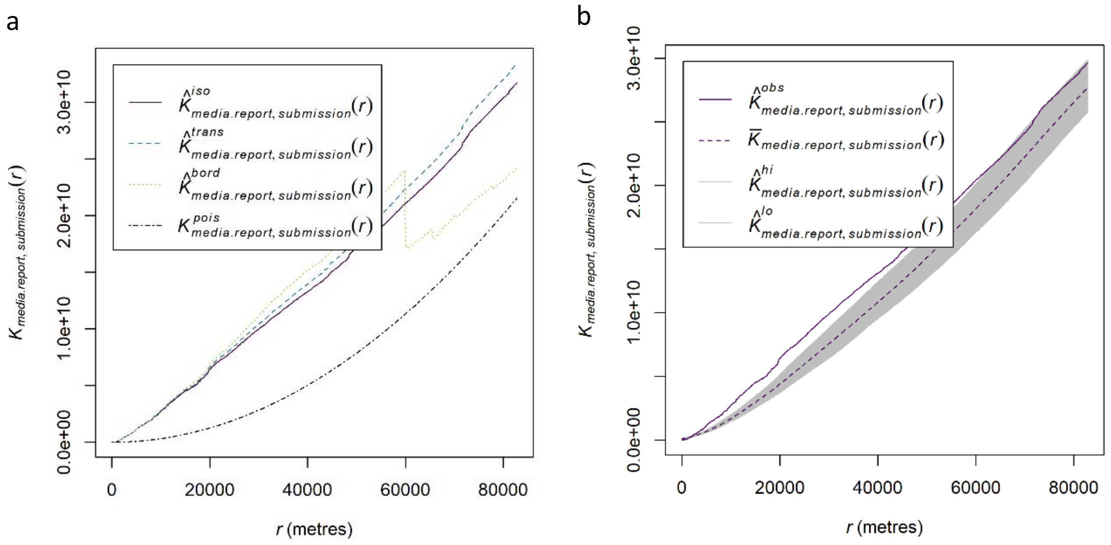

S3 Fig: Cross K-function and Monte Carlo simulation. (a) Bivariate Ripley's K function (Cross K-function) testing for similar clustering of media reports and submissions for distances up to 80 km. b) Cross K-function (black line) with simulation mean (dashed line) and significance bands (grey area) for random labelling applying a Monte Carlo simulation with 100 permutations.

S1 Table: List of variables of the media clipping dataset. Original variables (renamed) are highlighted in white and additionally programmed variables for analysis are highlighted in grey.

**Provided variables**	**Explanation**
Search terms (searcht)	Search term responsible for the hit
Publication date (date)	Exact date of media report
Headline (head)	Headline of the media report
Format (rad.tv)	Premiere or rerun
Link	URL to online media type
Media title (med.tit.cat)	Title of newspaper, radio or tv programme
Channel (med.ch)	Name of media channel (e.g. broadcaster of tv/radio show)
Media type (med.ty.cat)	Category of media (‘TV‘, ‘online‘, ‘print‘, ‘radio‘, ‘news agency‘)
Media category (med.ty.fine)	Refined media type (e.g. for TV documentary, news etc.)
**Additionally programmed variables**	
year	Year of media report
yearm	Year and month of media report
Municipality (med.muni)	Municipality of origin (see Supplementary Table S2)
xvalue	Longitude if municipality is given (see Supplementary Table S2)
yvalue	Latitude if municipality is given (see Supplementary Table S2)
Federal state (med.county)	Federal state of origin if applicable (see Supplementary Table S2)
Reach (reach)	Geographical reach (‘regional’, ‘single federal’, ‘cross federal’, ‘national’) (see Supplementary Table S2)

S2 Table: Categorisation method

In general, the categorisation and localisation of media contributions focused on the region to which they were addressed. A large part of the individual media were unknown, so that the distribution or broadcasting area was determined with the help of the Internet.

Case 1: media with national reach

National television, print and radio stations (e.g. *ARD*, *ZDF*, *Deutschlandfun*k) and general websites (e.g. *gmx*, *1&1*, *zeit online*). In these cases, no geo-coordinates were created for media reports.

Categorisation:

- Variable: Reach = ”national”
- Variable: Municipality = ”national”
- Variable: Federal state = ”national”

Case 2: media with reach over more than one federal state

Radio and television stations with an broadcasting area of more than one federal state, and some websites targeting several federal counties (e.g. *rbb*). In these cases, no geo-coordinates were created for media reports.

Categorisation:

- Variable: Reach = ”cross federal”
- Variable: Municipality = ”federal”
- Variable: Federal state = ”several”

Case 3: media targeting a single federal state or smaller region

Predominantly regional media, radio and television stations of individual federal states (e.g. *Die Glocke*, *WDR 2*, *BR Fernsehen*), and some websites aiming at a regional audience (e.g. *leinetal24.de*). If the distribution/broadcasting area concerned a city with its suburbs (e.g., *Augsburger Allgemeine*) or fewer than five municipalities (e.g. *Oberhessische Zeitung*), the geographic coordinates of the headquarters of the editorial office were used as the location for the corresponding media report. In addition, the name of the municipality was assigned to the media report in the category "Municipality" and the reach was set to “regional”. If the distribution/broadcasting area concerned more than one city and several regions (e.g. "Ostsee-Zeitung", WDR), no geo-location was assigned and the category "Municipality" was set to "federal". In both cases the name of the respective federal state was listed in the category "Federal state".

Categorisation:

- Variable: Reach = “regional” or ”single federal”
- Variable: Municipality = #respective name of the municipality or “federal”
- Variable: Federal state = #respective name of federal state

Below is the table with the respective assignments of the media reports to the categories. The number of formats is less than the number of all media reports, since the formats are sometimes doubled.

Media report title	Media channel	Reach	Municipality	Federal state
ARD-Buffet	ARD Arbeitsgemeinschaft der öffentlich-rechtlichen Rundfunkanstalten der Bundesrepublik Deutschland	cross federal	federal	several
ARD Infonacht	MDR Aktuell	cross federal	federal	several
ARD Infonacht	SWR Aktuell	cross federal	federal	several
Auf ein Wort	NDR Info	cross federal	federal	several
Bild Berlin-Brandenburg	NA	cross federal	federal	several
Das Informationsprogramm	NDR Info	cross federal	federal	several
DASDING	DASDING (SWR)	cross federal	federal	several
Die Profis	radioeins	cross federal	federal	several
Die Welt in 60 Sekunden	94,3 rs2 Berlin-Brandenburg Radio	cross federal	federal	several
Frankenschau aktuell	Bayerisches Fernsehen Nord	cross federal	federal	several
Hallo Niedersachsen	NDR Niedersachsen	cross federal	federal	several
Hallo Niedersachsen (Mo-So 11:00)	NDR Norddeutscher Rundfunk Anstalt des öffentlichen Rechts	cross federal	federal	several
Hauptsache gesund	MDR Mitteldeutscher Rundfunk - Anstalt des Öffentlichen Rechts	cross federal	federal	several
HNA Hessische Niedersächsische Allgemeine	NA	cross federal	federal	several
inFranken.de	NA	cross federal	federal	several
JUMP	MDR JUMP	cross federal	federal	several
Kreiszeitung.de	NA	cross federal	federal	several
Länder Eins30	MDR Mitteldeutscher Rundfunk - Anstalt des Öffentlichen Rechts	cross federal	federal	several
Lausitzer Rundschau	NA	cross federal	federal	several
Logo	NDR Info	cross federal	federal	several
Main-Echo	NA	cross federal	federal	several
Main-Spitze	NA	cross federal	federal	several
MDR	MDR Mitteldeutscher Rundfunk - Anstalt des Öffentlichen Rechts	cross federal	federal	several
MDR aktuell	MDR Mitteldeutscher Rundfunk - Anstalt des Öffentlichen Rechts	cross federal	federal	several
MDR JUMP	NA	cross federal	federal	several
MDR um 11	MDR Mitteldeutscher Rundfunk - Anstalt des Öffentlichen Rechts	cross federal	federal	several
MDR um 2	MDR Mitteldeutscher Rundfunk - Anstalt des Öffentlichen Rechts	cross federal	federal	several
Mitteldeutscher Rundfunk MDR	NA	cross federal	federal	several
Nachrichten 06:00	94,3 rs2 Berlin-Brandenburg Radio	cross federal	federal	several
Nachrichten 10:00	BB Radio Länderwelle Berlin/Brandenburg GmbH & Co. KG	cross federal	federal	several
Nachrichten 13:00	BB Radio Länderwelle Berlin/Brandenburg GmbH & Co. KG	cross federal	federal	several
Nachrichten 14:00	SWR Aktuell	cross federal	federal	several
Nachrichten 15:00	Spreeradio 105,5	cross federal	federal	several
Nachrichten 15:00	SWR 2	cross federal	federal	several
Nachrichten 15:00	SWR Aktuell	cross federal	federal	several
Nachrichten 17:00	Spreeradio 105,5	cross federal	federal	several
Nachrichtenradio	MDR Aktuell	cross federal	federal	several
NDR	NA	cross federal	federal	several
nordbuzz	NA	cross federal	federal	several
Nordmagazin	NDR Norddeutscher Rundfunk Anstalt des öffentlichen Rechts	cross federal	federal	several
OZON unterwegs	RBB Rundfunk Berlin Brandenburg Anstalt des öffentlichen Rechts	cross federal	federal	several

Planet Wissen	RBB Rundfunk Berlin Brandenburg Anstalt des öffentlichen Rechts	cross federal	federal	several
Prignitzer.de	NA	cross federal	federal	several
radioeins (16:00-19:00)	radioeins	cross federal	federal	several
rbb aktuell	RBB Rundfunk Berlin Brandenburg Anstalt des öffentlichen Rechts	cross federal	federal	several
rbb Rundfunk Berlin-Brandenburg	NA	cross federal	federal	several
rbb um 4	RBB Rundfunk Berlin Brandenburg Anstalt des öffentlichen Rechts	cross federal	federal	several
rbb um Sechs	RBB Rundfunk Berlin Brandenburg Anstalt des öffentlichen Rechts	cross federal	federal	several
Reportagen, Hintergründe, Interviews	SWR Aktuell	cross federal	federal	several
RTL Aktuell	104.6 RTL	cross federal	federal	several
rs2	94,3 rs2 Berlin-Brandenburg Radio	cross federal	federal	several
Sachsen-Anhalt heute	MDR Mitteldeutscher Rundfunk - Anstalt des Öffentlichen Rechts	cross federal	federal	several
SachsenSpiegel	MDR Mitteldeutscher Rundfunk - Anstalt des Öffentlichen Rechts	cross federal	federal	several
Standpunkte	NDR Info	cross federal	federal	several
SPUTNIK News 15:00	MDR Sputnik	cross federal	federal	several
SWR2 Aktuell mit Nachrichten 18:00	SWR 2	cross federal	federal	several
SWR2 Impuls	SWR 2	cross federal	federal	several
SWR2 Matinee	SWR 2	cross federal	federal	several
SWRinfo	SWR Aktuell	cross federal	federal	several
Tagesgespräch	Bayern 2	cross federal	federal	several
Umschau (MDR)	MDR Mitteldeutscher Rundfunk - Anstalt des Öffentlichen Rechts	cross federal	federal	several
Umschau (MDR)	NA	cross federal	federal	several
Umschau (Mi 00:30)	Tagesschau24	cross federal	federal	several
Unser Land	BR Bayerischer Rundfunk Anstalt des öffentlichen Rechtes	cross federal	federal	several
WDR 2	NA	cross federal	federal	several
Wochenkurier.info	NA	cross federal	federal	several
zibb	RBB Rundfunk Berlin Brandenburg Anstalt des öffentlichen Rechts	cross federal	federal	several
1&1	NA	national	national	national
2 Die Zwei	NA	national	national	national
3sat-Sendung	3sat	national	national	national
AD HOC NEWS	NA	national	national	national
alverde	NA	national	national	national
APA Science	NA	national	national	national
apotheke-adhoc.de	NA	national	national	national
Apotheken Magazin	NA	national	national	national
ARCOR	NA	national	national	national
ARD-Morgenmagazin	ARD Arbeitsgemeinschaft der öffentlich-rechtlichen Rundfunkanstalten der Bundesrepublik Deutschland	national	national	national
ARD-Morgenmagazin	Tagesschau24	national	national	national
ARD-Morgenmagazin	ZDF Zweites Deutsches Fernsehen Anstalt des öffentlichen Rechts	national	national	national
ARD Infonacht	InfoRadio	national	national	national
az-online.de	NA	national	national	national
AZV Anzeigenzeitungsverlag	NA	national	national	national
Baden Online	NA	national	national	national

bild der wissenschaft online	NA	national	national	national
Bild.de	NA	national	national	national
Bildwoche	NA	national	national	national
blog35215	NA	national	national	national
boote-forum.de	NA	national	national	national
Business Insider Deutschland	NA	national	national	national
Codecheck.info	NA	national	national	national
Das Kraftfuttermischwerk	NA	national	national	national
Deutschland heute	Deutschlandfunk	national	national	national
Deutschlandfunk	Deutschlandfunk	national	national	national
Deutschlandradio Kultur	Deutschlandfunk Kultur	national	national	national
Deutschlandradio Kultur	NA	national	national	national
Die PTA	NA	national	national	national
Die Reportage	Deutschlandfunk Kultur	national	national	national
DIE WELT	NA	national	national	national
Die Welt überregional	NA	national	national	national
drehscheibe Deutschland	ZDF Zweites Deutsches Fernsehen Anstalt des öffentlichen Rechts	national	national	national
Einsfestival-Reportage	Einsfestival	national	national	national
Einsfestival-Sendung	Einsfestival	national	national	national
esanum	NA	national	national	national
europe online magazine	NA	national	national	national
FAZ.net	NA	national	national	national
FOCUS Online	NA	national	national	national
Forschung aktuell	Deutschlandfunk	national	national	national
Frankfurter Neue Presse	NA	national	national	national
Frankfurter Rundschau	NA	national	national	national
Frühstücksfernsehen	SAT.1 Satelliten Fernsehen GmbH	national	national	national
Funk Uhr	NA	national	national	national
Galileo	ProSieben Television GmbH	national	national	national
gesund24	NA	national	national	national
GMX	NA	national	national	national
GreenTech Germany	NA	national	national	national
Grünstreifen	Deutschlandfunk Nova	national	national	national
Gutefrage.net	NA	national	national	national
Guten Morgen Deutschland	RTL Television GmbH	national	national	national
Guter Rat	NA	national	national	national
Heilpraxisnet.de	NA	national	national	national
Homepage - Galileo.tv - das Online-Wissensmagazin	NA	national	national	national
idowa.de	NA	national	national	national
idw Informationsdienst Wissenschaft	NA	national	national	national
InfoRadio	InfoRadio	national	national	national
innovations-report.de	NA	national	national	national
Linda magazin	NA	national	national	national
Mikroskopie Forum - Index	NA	national	national	national
MittelstandsWiki	NA	national	national	national
n-tv	NA	national	national	national

N24	NA	national	national	national
N24 Nachrichten	N24 Gesellschaft für Nachrichten und Zeitgeschehen mbH	national	national	national
Nachrichten 00:00-01:00	VOX Television GmbH	national	national	national
Nachrichten 06:00-09:00	n-tv Nachrichtenfernsehen GmbH	national	national	national
Nachrichten 09:00-10:00	n-tv Nachrichtenfernsehen GmbH	national	national	national
Neues Deutschland Bundesausgabe	NA	national	national	national
news.de	NA	national	national	national
news4kids	NA	national	national	national
ONE-Reportage	ONE	national	national	national
Onetz	NA	national	national	national
op-online.de	NA	national	national	national
OZON unterwegs	ARD-alpha	national	national	national
OZON unterwegs	EinsPlus	national	national	national
P.M. Magazin	NA	national	national	national
Pharmazeutische Zeitung online	NA	national	national	national
Pipeline	NA	national	national	national
planet e.	ZDF Zweites Deutsches Fernsehen Anstalt des öffentlichen Rechts	national	national	national
Planet Wissen	ARD-alpha	national	national	national
proplanta	NA	national	national	national
Punkt Zwölf	RTL Television GmbH	national	national	national
Redaktionskonferenz	Deutschlandfunk Nova	national	national	national
RTL Aktuell	RTL Television GmbH	national	national	national
RTL Nachtjournal	RTL Television GmbH	national	national	national
RTL West für Nordrhein-Westfalen	RTL West GmbH	national	national	national
Schattenblick	NA	national	national	national
Sonnenseite	NA	national	national	national
Spiegel Online	NA	national	national	national
Springer GuP	NA	national	national	national
Stern.de	NA	national	national	national
Süddeutsche.de	NA	national	national	national
Super TV	NA	national	national	national
SUPERillu	NA	national	national	national
T-Online	NA	national	national	national
Tagesschau-Nachrichten	Tagesschau24	national	national	national
Technology Review (Heise)	NA	national	national	national
The Epoch Times Deutschland	NA	national	national	national
Top Agrar	NA	national	national	national
Trierischer Volksfreund	NA	national	national	national
TV Gesund & Leben	NA	national	national	national
TV piccolino	NA	national	national	national
VBIO	NA	national	national	national
vetconsult	NA	national	national	national
VOX-Sendung	VOX Television GmbH	national	national	national
Web Nachrichten	NA	national	national	national
WEB.DE	NA	national	national	national

Welt am Sonntag überregional	NA	national	national	national
Welt der Wunder	N24 Gesellschaft für Nachrichten und Zeitgeschehen mbH	national	national	national
WIRED	NA	national	national	national
Wissensnachrichten	Deutschlandfunk Nova	national	national	national
X:enius	Arte G.E.I.E.	national	national	national
Yahoo! Nachrichten	NA	national	national	national
Zeit Online	NA	national	national	national
Aachener Nachrichten	NA	regional	Aachen	North Rhine-Westphalia
Aachener Zeitung	NA	regional	Aachen	North Rhine-Westphalia
Abendschau	Bayerisches Fernsehen Süd	single federal	federal	Bavaria
Abendschau	RBB Berlin	single federal	federal	Berlin
Abendschau	RBB Rundfunk Berlin Brandenburg Anstalt des öffentlichen Rechts	single federal	federal	Berlin
Abendzeitung München	NA	regional	Munich	Bavaria
aktuell	a.tv GmbH & Co. KG	regional	Augsburg	Bavaria
Aktuell (Tageszusammenfassung)	NDR 1 Niedersachsen	single federal	federal	Lower Saxony
Aktuell am Abend	SWR Aktuell	single federal	federal	Baden-Württemberg
Alle Wetter	Hessischer Rundfunk - Anstalt des öffentlichen Rechts	single federal	federal	Hesse
Allgäuer Zeitung Kempten	NA	regional	Kempten	Bavaria
Allgemeine Zeitung (Rhein Main Presse)	NA	single federal	federal	Hesse
Allgemeine Zeitung Coesfeld	NA	regional	Coesfeld	North Rhine-Westphalia
Allgemeine Zeitung Mainz	NA	regional	Mainz	Rhineland-Palatinate
Alsfelder Allgemeine	NA	regional	Alsfeld	Hesse
Altmark Zeitung Salzwedeler Nachrichten	NA	regional	Salzwedel	Saxony-Anhalt
Am Nachmittag	SWR 4 Baden-Württemberg	single federal	federal	Baden-Württemberg
Antenne-Gespräch	Antenne Brandenburg	single federal	federal	Brandenburg
Antenne Brandenburg	Antenne Brandenburg	single federal	federal	Brandenburg
antenne unna	NA	regional	Unna	North Rhine-Westphalia
ARD-Buffet	SWR Baden-Württemberg	single federal	federal	Baden-Württemberg
ARD-Buffet	RBB Rundfunk Berlin Brandenburg Anstalt des öffentlichen Rechts	single federal	federal	Berlin
ARD-Buffet	SWR Rheinland-Pfalz	single federal	federal	Rhineland-Palatinate
ARD-Buffet	Saarländischer Rundfunk	single federal	federal	Saarland
ARD Infonacht	B5 aktuell	single federal	federal	Bavaria
Augsburger Allgemeine	NA	regional	Augsburg	Bavaria
Augsburger Allgemeine	NA	regional	Augsburg	Berlin
Augsburger Allgemeine AS	NA	regional	Augsburg	Bavaria
B.Z.	NA	regional	Berlin	Berlin
B5 aktuell	B5 aktuell	single federal	federal	Bavaria
Bad Vilbeler Neue Presse	NA	regional	Bad Vilbel	Hesse
Badische Neueste Nachrichten ST	NA	regional	Karlsruhe	Baden-Württemberg
Badische Zeitung	NA	regional	Freiburg	Baden-Württemberg
Badisches Tagblatt	NA	regional	Baden-Baden	Baden-Württemberg
Bayerische Rundschau	NA	regional	Kulmbach	Bavaria
Bayern 1 am Vormittag	Bayern 1	single federal	federal	Bavaria

BAYERN 3 - Update	Bayern 3	single federal	federal	Bavaria
Bayern und die Welt	Antenne Bayern	single federal	federal	Bavaria
Bayern Update um 12:30	Antenne Bayern	single federal	federal	Bavaria
Berlin Report 11:30	Radio Berlin 88,8	regional	Berlin	Berlin
Berlin.de	NA	regional	Berlin	Berlin
Berliner Kurier	NA	regional	Berlin	Berlin
Berliner Morgenpost	NA	regional	Berlin	Berlin
Berliner Zeitung	NA	regional	Berlin	Berlin
BerlinOnline	NA	regional	Berlin	Berlin
BKZ Online	NA	regional	Backnang	Baden-Württemberg
BlickPunkt Nienburg	NA	regional	Nienburg	Lower Saxony
Bocholter-Borkener Volksblatt	NA	regional	Bocholt	North Rhine-Westphalia
Böhme-Zeitung	NA	regional	Soltau	Lower Saxony
bonus	Saarländischer Rundfunk	single federal	federal	Saarland
Borkener Zeitung	NA	regional	Borken	North Rhine-Westphalia
BR.de - Bayerischer Rundfunk	NA	single federal	federal	Bavaria
BR.de Bayerisches Fernsehen	NA	single federal	federal	Bavaria
Brandenburg aktuell	RBB Brandenburg	single federal	federal	Brandenburg
Brandenburg aktuell	RBB Rundfunk Berlin Brandenburg Anstalt des öffentlichen Rechts	single federal	federal	Brandenburg
Braunschweiger Zeitung	NA	regional	Braunschweig	Lower Saxony
Bremer Nachrichten Die Norddeutsche	NA	regional	Bremen	Bremen
Bürstädter Zeitung	NA	regional	Bürstadt	Hesse
Cellesche Zeitung	NA	regional	Celle	Lower Saxony
Chemnitzer Morgenpost	NA	regional	Chemnitz	Saxony
chiemgau24.de	NA	single federal	federal	Bavaria
CityNEWS	NA	regional	Cologne	North Rhine-Westphalia
come-on.de	NA	single federal	federal	North Rhine-Westphalia
DA-imNetz.de (Dieburger Anzeiger)	NA	regional	Dieburg	Hesse
daheim + unterwegs	WDR Westdeutscher Rundfunk Anstalt des öffentlichen Rechts	single federal	federal	North Rhine-Westphalia
Darmstädter Echo	NA	regional	Darmstadt	Hesse
Das Gelbe Blatt	NA	regional	Penzberg	Bavaria
Der gute Morgen	Bremen Zwei	regional	Bremen	Bremen
Der Morgen	Domradio	regional	Cologne	North Rhine-Westphalia
Der Nachmittag	MDR 1 Radio Sachsen	single federal	federal	Saxony
Der Neue Tag Weiden	NA	regional	Weiden	Bavaria
Der Prignitzer Brandenburger Allgemeine	NA	single federal	federal	Brandenburg
Der Tag um Vier	NDR 1 Radio MV	single federal	federal	Mecklenburg-Western Pomerania
Der Tagesspiegel	NA	regional	Berlin	Berlin
DEWEZET Deister-und Weserzeitung	NA	regional	Hameln	North Rhine-Westphalia
DEWEZET.de	NA	regional	Hameln	North Rhine-Westphalia
Die besten Schlager und Oldies (14 - 19)	HR4	single federal	federal	Hesse
Die Glocke	NA	regional	Oelde	North Rhine-Westphalia

Die Oberbadische	NA	regional	Lörrach	Baden-Württemberg
Dieburger Anzeiger	NA	regional	Dieburg	Hesse
Dresdner Neueste Nachrichten	NA	regional	Dresden	Saxony
Dülmener Zeitung	NA	regional	Dülmen	North Rhine-Westphalia
Echo Online	NA	regional	Darmstadt	Hesse
echo24.de	NA	regional	Heilbronn	Baden-Württemberg
Ehinger Tagblatt	NA	regional	Ehingen	Baden-Württemberg
eisenberg.TLZ.de	NA	regional	Eisenberg	Thuringia
Elbe-Jeetzel-Zeitung	NA	regional	Lüchow	Lower Saxony
Elbe-Jeetzel-Zeitung online	NA	regional	Lüchow	Lower Saxony
Emder Zeitung	NA	regional	Emden	Lower Saxony
Express Köln	NA	regional	Cologne	North Rhine-Westphalia
Express.de	NA	regional	Cologne	North Rhine-Westphalia
fehmarn 24	NA	single federal	federal	Schleswig-Holstein
Flensburger Tageblatt	NA	regional	Flensburg	Schleswig-Holstein
FluxFM aktuell 15:00	FluxFM	regional	Berlin	Berlin
FluxFM aktuell 16:00	FluxFM	regional	Berlin	Berlin
FORUM (Freising)	NA	regional	Freising	Bavaria
Frankenpost	NA	regional	Hof	Bavaria
Fränkische Nachrichten	NA	regional	Tauberbischofsheim	Bavaria
Fränkischer Tag	NA	regional	Bamberg	Bavaria
Freie Presse Chemnitzer Zeitung	NA	regional	Chemnitz	Saxony
freiepresse.de	NA	single federal	federal	Saxony
Freies Wort Suhl	NA	regional	Suhl	Thuringia
Fuldaer Zeitung	NA	regional	Fulda	Hesse
Gäubote	NA	regional	Herrenberg	Baden-Württemberg
Gelnhäuser Tageblatt	NA	regional	Gelnhausen	Hesse
General-Anzeiger Bonn	NA	regional	Bonn	North Rhine-Westphalia
Gießener Allgemeine	NA	regional	Gießen	Hesse
Gießener Anzeiger	NA	regional	Gießen	Hesse
GMÜNDER TAGESPOST	NA	regional	Schwäbisch Gmünd	Baden-Württemberg
Goslarsche Zeitung	NA	regional	Goslar	Lower Saxony
Göttinger Tageblatt	NA	regional	Göttingen	Lower Saxony
Guten Morgen Sachsen	MDR 1 Radio Sachsen	single federal	federal	Saxony
Haller Kreisblatt	NA	regional	Halle	Saxony-Anhalt
Hallo Brandenburg	Antenne Brandenburg	single federal	federal	Brandenburg
Hallo München	NA	regional	Munich	Bavaria
Hamburg "Der Morgen"	NDR 90,3	regional	Hamburg	Hamburg
Hamburger Abendblatt	NA	regional	Hamburg	Hamburg
Hamburger Morgenpost	NA	regional	Hamburg	Hamburg
Hannoversche Allgemeine Zeitung	NA	regional	Hannover	Lower Saxony
Harz Kurier Herzberger Zeitung	NA	regional	Herzberg	Lower Saxony
Heidelberg24	NA	regional	Heidelberg	Baden-Württemberg
Heilbronner Stimme	NA	regional	Heilbronn	Baden-Württemberg

heimatzeitung.de	NA	single federal	federal	Bavaria
Hellweger Anzeiger Unna	NA	regional	Unna	North Rhine-Westphalia
Hersfelder Zeitung	NA	regional	Hersfeld	Hesse
Hessen & die Welt	Hitradio FFH	single federal	federal	Hesse
Hessisch Niedersächsische Allgemeine	NA	single federal	federal	Hesse
Hildesheimer Allgemeine Zeitung	NA	regional	Hildesheim	Lower Saxony
Höchster Kreisblatt	NA	regional	Höchst	Hesse
hr-iNFO	HR Info	single federal	federal	Hesse
hr1	HR1	single federal	federal	Hesse
hr1	NA	single federal	federal	Hesse
Ihr Vormittag	Radio Berlin 88,8	regional	Berlin	Berlin
IKZ - Iserlohner Kreisanzeiger	NA	regional	Iserlohn	North Rhine-Westphalia
in Südthüringen.de	NA	single federal	federal	Thuringia
innsalzach24.de	NA	single federal	federal	Bavaria
IQ - Wissenschaft und Forschung	Bayern 2	single federal	federal	Bavaria
Kieler Nachrichten	NA	regional	Kiel	Schleswig-Holstein
Kölnische Rundschau	NA	regional	Cologne	North Rhine-Westphalia
Kompakt	Domradio	regional	Cologne	North Rhine-Westphalia
Kreis-Anzeiger (Wetterau)	NA	regional	Friedberg	Hesse
Kreisbote	NA	single federal	federal	Bavaria
Kreiszeitung Böblinger Bote	NA	regional	Böblingen	Baden-Württemberg
Kreiszeitung Syker Zeitung	NA	regional	Syke	Lower Saxony
Kreiszeitung Wesermarsch	NA	regional	Nordenham	Lower Saxony
Kurierverlag	NA	regional	Memmingen	Bavaria
Lampertheimer Zeitung	NA	regional	Lampertheim	Hesse
Landeszeitung für die Lüneburger Heide	NA	regional	Lüneburg	Lower Saxony
Lauterbacher Anzeiger	NA	regional	Lauterbach	Hesse
leinetal24.de	NA	single federal	federal	Lower Saxony
Leipzig kompakt 18:20	Radio Leipzig	regional	Leipzig	Saxony
Leipziger Volkszeitung	NA	regional	Leipzig	Saxony
Leonardo: Wissenschaft und mehr	WDR 5	single federal	federal	North Rhine-Westphalia
Lippische Landeszeitung	NA	single federal	federal	North Rhine-Westphalia
LN Online - Lübecker Nachrichten	NA	regional	Lübeck	Schleswig-Holstein
Lokalzeit aus Bonn (Di-Sa 10:00)	WDR Bonn	regional	Bonn	North Rhine-Westphalia
Lokalzeit aus Bonn (Mo-Sa 6:20)	WDR Westdeutscher Rundfunk Anstalt des öffentlichen Rechts	regional	Bonn	North Rhine-Westphalia
Lokalzeit aus Bonn 19:30	WDR Bonn	regional	Bonn	North Rhine-Westphalia
LS aktuell Baden-Württemberg 18:00	SWR Baden-Württemberg	single federal	federal	Baden-Württemberg
Lünepost am Wochenende	NA	regional	Lüneburg	Lower Saxony
Magdeburger Volksstimme Magdeburgische Zeitung	NA	regional	Magdeburg	Saxony-Anhalt
Maintal Tagesanzeiger	NA	regional	Hanau	Hesse
Mannheim24	NA	regional	Mannheim	Baden-Württemberg
Märkische Allgemeine	NA	single federal	federal	Brandenburg

Märkische Allgemeine Potsdamer Tageszeitung	NA	regional	Potsdam	Brandenburg
Märkische Oderzeitung	NA	regional	Frankfurt an der Oder	Brandenburg
Märkische Oderzeitung Frankfurter Stadtbote	NA	regional	Frankfurt an der Oder	Brandenburg
MDR 1 Sachsen-Anhalt	MDR Radio Sachsen-Anhalt	single federal	federal	Saxony-Anhalt
MDR Thüringen	MDR Radio Thüringen	single federal	federal	Thuringia
Meininger Tageblatt	NA	regional	Meiningen	Thuringia
Merkur.de	NA	regional	Munich	Bavaria
Mindener Tageblatt	NA	regional	Minden	North Rhine-Westphalia
Mit WDR 2 in den Feierabend	WDR 2	single federal	federal	North Rhine-Westphalia
Mittagsschau kompakt 13:00	NDR 1 Radio MV	single federal	federal	Mecklenburg-Western Pomerania
Mittelbayerische Zeitung	NA	single federal	federal	Bavaria
Mittelbayerische Zeitung Tagesanzeiger	NA	single federal	federal	Bavaria
Mitteldeutsche Zeitung Halle / Saalkreis	NA	regional	Halle	Saxony-Anhalt
mittelhessen	NA	single federal	federal	Hesse
morgenweb	NA	regional	Mannheim	Baden-Württemberg
Münsterländische Volkszeitung	NA	regional	Rheine	North Rhine-Westphalia
Münstersche Zeitung Westfalen-Anzeiger	NA	regional	Münster	North Rhine-Westphalia
Münstersche Zeitung.de	NA	regional	Münster	North Rhine-Westphalia
Nachrichten 06:00	Neue Berliner Rundfunk GmbH & Co KG	regional	Berlin	Berlin
Nachrichten 06:00	Radio Charivari Würzburg	regional	Würzburg	Bavaria
Nachrichten 08:00	Neue Berliner Rundfunk GmbH & Co KG	regional	Berlin	Berlin
Nachrichten 08:00	Radio Charivari Würzburg	regional	Würzburg	Bavaria
Nachrichten 10:00	Neue Berliner Rundfunk GmbH & Co KG	regional	Berlin	Berlin
Nachrichten 11:00	Neue Berliner Rundfunk GmbH & Co KG	regional	Berlin	Berlin
Nachrichten 13:00	Radio Hamburg GmbH & Co. KG	regional	Hamburg	Hamburg
Nachrichten 14:00	Radio Hamburg GmbH & Co. KG	regional	Hamburg	Hamburg
Nachrichten 14:00	Radio Charivari Würzburg	regional	Würzburg	Bavaria
Nachrichten 14:55	Energy Bremen	regional	Bremen	Bremen
Nachrichten 15:00	radio B2	single federal	federal	Bavaria
Nachrichten 15:55	Energy Bremen	regional	Bremen	Bremen
Nachrichten 16:00	radio B2	single federal	federal	Bavaria
Nachrichten 16:00	Radio Charivari Würzburg	regional	Würzburg	Bavaria
Nachrichten 17:00	Radio Charivari Würzburg	regional	Würzburg	Bavaria
Nachrichten 18:00	WDR 2	single federal	federal	North Rhine-Westphalia
Nachrichten 18:00	WDR 5	single federal	federal	North Rhine-Westphalia
Nachrichten 19:00	SWR 4 Baden-Württemberg	single federal	federal	Baden-Württemberg
Nachrichten 19:00	SWR 4 Rheinland-Pfalz	single federal	federal	Rhineland-Palatinate
Nachrichten 19:53	Radio Gong 96,3	single federal	federal	Bavaria
Nachrichten 20:00	Radio Arabella Studiobetriebsges mbH	single federal	federal	Bavaria
Nachrichten 20:00	Radio Charivari Würzburg	regional	Würzburg	Bavaria
Nachrichten für Niedersachsen 18:00	radio ffn	single federal	federal	Lower Saxony
Nassauische Neue Presse	NA	regional	Nassau	Rhineland-Palatinate

NDR 1 Radio MV aktuell 22:00	NDR 1 Radio MV	single federal	federal	Mecklenburg-Western Pomerania
NDR1 Niedersachsen Aktuell 19:00	NDR 1 Niedersachsen	single federal	federal	Lower Saxony
NDZ.de Neue Deister Zeitung	NA	regional	Hameln	Lower Saxony
Netzwelt	HR Info	single federal	federal	Hesse
Neu-Isenburger Neue Presse	NA	regional	Neu-Isenburg	Hesse
Neue Osnabrücker Zeitung	NA	regional	Osnabrück	Lower Saxony
Neue Presse Coburg	NA	regional	Coburg	Bavaria
Neue Westfälische	NA	single federal	federal	North Rhine-Westphalia
Neue Westfälische Bielefelder Tageblatt OH	NA	regional	Bielefeld	North Rhine-Westphalia
Niedersachsen 18.00	NDR Niedersachsen	single federal	federal	Lower Saxony
nordbayern.de	NA	single federal	federal	Bavaria
Norddeutsche Neueste Nachrichten	NA	regional	Rostock	Mecklenburg-Western Pomerania
Nordkurier	NA	single federal	federal	Mecklenburg-Western Pomerania
Nordmagazin	NDR Mecklenburg-Vorpommern	single federal	federal	Mecklenburg-Western Pomerania
Nordwest-Zeitung	NA	regional	Oldenburg	Lower Saxony
NRZ - Neue Ruhr Zeitung	NA	single federal	federal	North Rhine-Westphalia
Nürnberger Nachrichten	NA	regional	Nürnberg	Bavaria
Nürtinger Zeitung	NA	regional	Nürtingen	Baden-Württemberg
NZ Nürnberger Zeitung NZS	NA	regional	Nürnberg	Bavaria
Oberhessische Presse	NA	regional	Marburg	Hesse
Oberhessische Zeitung	NA	regional	Alsfeld	Hesse
Offenbach-Post	NA	regional	Offenbach	Hesse
Oranienburger Generalanzeiger	NA	regional	Oranienburg	Brandenburg
Ostfriesische Nachrichten	NA	regional	Aurich	Lower Saxony
Ostsee-Zeitung	NA	single federal	federal	Mecklenburg-Western Pomerania
Ostseewelle HIT-RADIO Mecklenburg-Vorpommern	NA	single federal	federal	Mecklenburg-Western Pomerania
Ostthüringer Zeitung	NA	single federal	federal	Thuringia
OVB online (Oberbayerisches Volksblatt)	NA	single federal	federal	Bavaria
Panorama	Antenne Brandenburg	single federal	federal	Brandenburg
Pirmasenser Zeitung	NA	regional	Pirmasens	Rhineland-Palatinate
Planet Wissen	WDR Westdeutscher Rundfunk Anstalt des öffentlichen Rechts	single federal	federal	North Rhine-Westphalia
Planet Wissen	SWR Baden-Württemberg	single federal	federal	Baden-Württemberg
Planet Wissen	SWR Rheinland-Pfalz	single federal	federal	Rhineland-Palatinate
Planet Wissen	Saarländischer Rundfunk	single federal	federal	Saarland
Platzhalter Bayern 1 München	Bayern 1	single federal	federal	Bavaria
Platzhalter Radio NRW	Lokalradio Düsseldorf Betriebsgesellschaft mbH & Co. KG	regional	Düsseldorf	North Rhine-Westphalia
Potsdam am Sonntag	NA	regional	Potsdam	Brandenburg
Potsdamer Neueste Nachrichten	NA	regional	Potsdam	Brandenburg
Punkt	Radio Regenbogen Hörfunk in Baden GmbH & Co. KG	single federal	federal	Baden-Württemberg
Pyrmonter Nachrichten	NA	regional	Pyrmont	Lower Saxony
Radio B2	radio B2	single federal	federal	Bavaria

Radio Chemnitz	NA	regional	Chemnitz	Saxony
Radio SAW Online	NA	single federal	federal	Saxony-Anhalt
Recklinghäuser Zeitung	NA	regional	Recklinghausen	North Rhine-Westphalia
regio-news.de	NA	regional	Karlsruhe	Baden-Württemberg
Regional-News aus Potsdam	Antenne Brandenburg Potsdam	regional	Potsdam	Brandenburg
Regionaljournal	Antenne Brandenburg	single federal	federal	Brandenburg
regionalZeit - Südbayern	Bayern 2	single federal	federal	Bavaria
Remscheider General-Anzeiger	NA	regional	Remscheid	North Rhine-Westphalia
Reutlinger General-Anzeiger	NA	regional	Reutlingen	Baden-Württemberg
Rhein-Neckar-Zeitung	NA	regional	Heidelberg	Baden-Württemberg
Rhein-Zeitung	NA	single federal	federal	Rhineland-Palatinate
Rhein-Zeitung Koblenz	NA	regional	Koblenz	Rhineland-Palatinate
Rhein Main EXTRA TIPP	NA	single federal	federal	Hesse
RHEIN MAIN PRESSE	NA	single federal	federal	Hesse
Rheinische Post D Düsseldorf	NA	regional	Düsseldorf	North Rhine-Westphalia
rosenheim24.de	NA	regional	Rosenheim	Bavaria
Rotenburger Kreiszeitung	NA	regional	Rotenburg	Lower Saxony
RP-Online Rheinische Post	NA	single federal	federal	North Rhine-Westphalia
RTL Hessen	RTL Hessen GmbH	single federal	federal	Hesse
Ruhr Nachrichten Dortmunder Zeitung Süd (D1)	NA	regional	Dortmund	North Rhine-Westphalia
Rundschau-Magazin	BR Bayerischer Rundfunk Anstalt des öffentlichen Rechtes	single federal	federal	Bavaria
Rüsselsheimer Echo	NA	regional	Rüsselsheim	Hesse
Saale-Zeitung	NA	regional	Bad Kissingen	Bavaria
Sachsen-Anhalt heute	MDR Sachsen-Anhalt	single federal	federal	Saxony-Anhalt
SachsenSpiegel	MDR Sachsen	single federal	federal	Saxony
Sächsische Zeitung	NA	regional	Dresden	Saxony
Sächsische Zeitung - SZ-Online.de	NA	regional	Dresden	Saxony
Sauerland-Kurier	NA	regional	Olpe	North Rhine-Westphalia
SauerlandKurier	NA	regional	Olpe	North Rhine-Westphalia
Schaumburg-Lippische Landes-Zeitung.de	NA	regional	Schaumburg	Lower Saxony
Schaumburger Nachrichten	NA	regional	Schaumburg	Lower Saxony
Schaumburger Zeitung	NA	regional	Schaumburg	Lower Saxony
Schaumburger Zeitung.de	NA	regional	Schaumburg	Lower Saxony
Schwäbische Post	NA	single federal	federal	Bavaria
Schwäbische Zeitung	NA	single federal	federal	Bavaria
Schwäbische Zeitung Ravensburg	NA	regional	Ravensburg	Bavaria
Schweriner Volkszeitung Mecklenburgische Zeitung	NA	regional	Schwerin	Mecklenburg-Western Pomerania
Segeberger Zeitung	NA	regional	Segeberg	Schleswig-Holstein
Service: Gesundheit	Hessischer Rundfunk - Anstalt des öffentlichen Rechts	single federal	federal	Hesse
SHZ.de	NA	single federal	federal	Schleswig-Holstein
Siegener Zeitung	NA	regional	Siegen	North Rhine-Westphalia

Siegerland-Kurier	NA	regional	Siegen	North Rhine-Westphalia
Soester Anzeiger	NA	regional	Soest	North Rhine-Westphalia
Solinger Tageblatt	NA	regional	Solingen	North Rhine-Westphalia
Sonntagsjournal der Bremervörder Zeitung	NA	regional	Gnarrenburg	Lower Saxony
Sonntagsjournal der Nordsee-Zeitung	NA	single federal	federal	Lower Saxony
Sonntagsjournal der Zevener Zeitung	NA	regional	Zeven	Lower Saxony
Stuttgarter Nachrichten	NA	regional	Stuttgart	Baden-Württemberg
Stuttgarter Zeitung	NA	regional	Stuttgart	Baden-Württemberg
Stuttgarter Zeitung D	NA	regional	Stuttgart	Baden-Württemberg
STZ Südthüringer Zeitung Bad Salzungen	NA	regional	Bad Salzungen	Thuringia
Südwest Presse	NA	regional	Ulm	Baden-Württemberg
SVZ.de Schweriner Volkszeitung	NA	regional	Schwerin	Mecklenburg-Western Pomerania
Taunus Zeitung	NA	regional	Bad Homburg	Hesse
Thüringer Allgemeine	NA	regional	Erfurt	Thuringia
Thüringische Landeszeitung	NA	regional	Erfurt	Thuringia
tv.berlin Aktuell	TV Berlin	regional	Berlin	Berlin
TZ online	NA	regional	Berlin	Berlin
Uckermark Kurier	NA	single federal	federal	Brandenburg
Usinger Anzeiger	NA	regional	Usingen	Hesse
volksfreund.de	NA	regional	Trier	Rhineland-Palatinate
WA.de - Westfälischer Anzeiger	NA	regional	Hamm	North Rhine-Westphalia
Waldeckische Landeszeitung	NA	regional	Waldeck	Hesse
Walsroder Zeitung	NA	regional	Walsrode	Lower Saxony
Waltroper Zeitung	NA	regional	Waltrop	North Rhine-Westphalia
WAZ - Westdeutsche Allgemeine Zeitung	NA	single federal	federal	North Rhine-Westphalia
WDR 2 am Sonntag (09:00-14:00)	WDR 2	single federal	federal	North Rhine-Westphalia
WDR 2 Servicezeit	WDR 2	single federal	federal	North Rhine-Westphalia
WDR 3 Resonanzen	WDR 3	single federal	federal	North Rhine-Westphalia
Werra-Rundschau	NA	regional	Eschwege	Hesse
Weser Kurier Bremer Tageszeitung	NA	regional	Bremen	Bremen
Westdeutsche Zeitung	NA	regional	Wuppertal	North Rhine-Westphalia
Westdeutscher Rundfunk	NA	regional	Wuppertal	North Rhine-Westphalia
Westfälische Nachrichten	NA	regional	Münster	North Rhine-Westphalia
Westfälische Nachrichten Münsterischer Anzeiger	NA	regional	Münster	North Rhine-Westphalia
Wetterauer Zeitung	NA	regional	Friedberg	Hesse
Wetzlarer Neue Zeitung	NA	regional	Wetzlar	Hesse
Wiesbadener Kurier	NA	regional	Wiesbaden	Hesse
Wiesbadener Tagblatt	NA	regional	Wiesbaden	Hesse
Wilhelmshavener Zeitung	NA	regional	Wilhelmshaven	Lower Saxony
Winsener Anzeiger	NA	regional	Winsen	Lower Saxony

Wolfsburger Allgemeine	NA	regional	Wolfsburg	Lower Saxony
Wormser Zeitung	NA	regional	Worms	Rhineland-Palatinate
WP - Westfalenpost	NA	regional	Hagen	North Rhine-Westphalia
WR - Westfälische Rundschau	NA	single federal	federal	North Rhine-Westphalia
WR Westfälische Rundschau Kamen, Bergkamen	NA	regional	Kamen	North Rhine-Westphalia
WZ Westdeutsche Zeitung Düsseldorf	NA	regional	Düsseldorf	North Rhine-Westphalia
Zevener Zeitung	NA	regional	Zeven	Lower Saxony
Zollern-Alb Kurier	NA	regional	Balingen	Baden-Württemberg

S3 Table: Annual numbers of media reports and submissions

Year	Media reports	Submissions
2014	31	1903
2015	66	1221
2016	542	7756
2017	295	5730

S4 Table: Headlines of the Berlin/Brandenburg office dpa-releases used for qualitative analysis. The clipping service sometimes recorded a dpa-release several times in one day, the number of these duplications are enclosed in brackets. Daily previews announcing dpa-releases were not included.

**dpa-release headline (in German)**	**Release date**
Auch Mücken und Nacktschnecken lieben den Start-up-Sommer (2)	20.05.2014
Sommerstart auch für Mücken und Nacktschnecken	20.05.2014
Mückenatlas: Forscher fahnden nach neuen Stechmückenarten	25.05.2015
Biologin: Mückensaison bislang lau - «Ohne Wasser keine Mücken»	08.08.2015
Biologin: Mückensaison bislang lau - «Ohne Wasser keine Mücken»	09.08.2015
Beißen und Stechen - Welche Tiere im Sommer besonders nerven	14.08.2015
Jede Mücke zählt - Wie sich Exoten in Deutschland etablieren	16.11.2015
2015 war kein Mückenjahr - Weniger Einsendungen für den Mückenatlas	16.11.2015
Sommer, Sonne, Mücke - Plagegeister surren wieder	23.05.2016
Verdächtiges Surren: Mücken fliegen wieder	23.05.2016
**Forscher-Bitte: Bürger sollen Mücken schicken**	**06.06.2016**
**Mückenplage droht - Bürger sollen Exemplare einschicken (3)**	**06.06.2016**
Mückenforscher freuen sich über so viel Post wie nie zuvor	02.11.2016
Mückenjäger fangen 30 000 Tiere für die Forschung	02.11.2016
Mückenforscherin kartiert die kleinen Plagegeister	03.02.2017
33 500 Mücken und noch lange kein Ende	03.02.2017
Sie sind schon da: Sonniger Frühling lässt Mücken eher ausschwärmen	10.04.2017
Stechende Plagegeister - warmer Frühsommer gut für Mücken	03.06.2017
Surren und Stechen: Warmer Frühsommer ist für Mücken ideal	03.06.2017
Surren und Stechen: Warmer Frühsommer ist für Mücken ideal	05.06.2017
Nach dem Regen: Hochsaison für Mücken	22.07.2017
Expertin zur Mückenplage	22.07.2017
Das große Surren - alle zwei Wochen neue Mückengeneration	22.07.2017
Expertin zur Mückenplage	23.07.2017
Das große Surren - alle zwei Wochen neue Mückengeneration	23.07.2017
Sommer 2017 ist ideal für Mücken und schlecht für Wespen	28.07.2017
«Die Mücken schreien hurra» - ideale Bedingungen nach Regenflut	28.07.2017
«Mücke tobt überall» - Nahender Herbst treibt Hausmücken in Verstecke	07.09.2017
«Es ist die Hölle»: Nahender Herbst treibt Stechmücken ins Haus	07.09.2017
